# Oral Pathobiont-Induced Changes in Gut Microbiota Aggravate the Pathology of Nonalcoholic Fatty Liver Disease in Mice

**DOI:** 10.3389/fimmu.2021.766170

**Published:** 2021-10-11

**Authors:** Kyoko Yamazaki, Tamotsu Kato, Yuuri Tsuboi, Eiji Miyauchi, Wataru Suda, Keisuke Sato, Mayuka Nakajima, Mai Yokoji-Takeuchi, Miki Yamada-Hara, Takahiro Tsuzuno, Aoi Matsugishi, Naoki Takahashi, Koichi Tabeta, Nobuaki Miura, Shujiro Okuda, Jun Kikuchi, Hiroshi Ohno, Kazuhisa Yamazaki

**Affiliations:** ^1^ Research Unit for Oral-Systemic Connection, Division of Oral Science for Health Promotion, Niigata University Graduate School of Medical and Dental Sciences, Niigata, Japan; ^2^ Division of Periodontology, Niigata University Graduate School of Medical and Dental Sciences, Niigata, Japan; ^3^ Laboratory for Intestinal Ecosystem, RIKEN Centre for Integrative Medical Sciences (IMS), Yokohama, Japan; ^4^ RIKEN Center for Sustainable Resource Science, Yokohama, Japan; ^5^ Laboratory for Microbiome Sciences, RIKEN Center for Integrative Medical Sciences, Yokohama, Japan; ^6^ Division of Bioinformatics, Niigata University Graduate School of Medical and Dental Sciences, Niigata, Japan; ^7^ Medical AI Center, Niigata University School of Medicine, Niigata, Japan; ^8^ Intestinal Microbiota Project, Kanagawa Institute of Industrial Science and Technology, Kawasaki, Japan

**Keywords:** metabolome, metagenomic analysis, NAFLD, periodontopathic bacteria, oral–gut connection, periodontitis, *Porphyromonas gingivalis*

## Abstract

**Background & Aims:**

Periodontitis increases the risk of nonalcoholic fatty liver disease (NAFLD); however, the underlying mechanisms are unclear. Here, we show that gut dysbiosis induced by oral administration of *Porphyromonas gingivalis*, a representative periodontopathic bacterium, is involved in the aggravation of NAFLD pathology.

**Methods:**

C57BL/6N mice were administered either vehicle, *P. gingivalis*, or *Prevotella intermedia*, another periodontopathic bacterium with weaker periodontal pathogenicity, followed by feeding on a choline-deficient, l-amino acid-defined, high-fat diet with 60 kcal% fat and 0.1% methionine (CDAHFD60). The gut microbial communities were analyzed by pyrosequencing the 16S ribosomal RNA genes. Metagenomic analysis was used to determine the relative abundance of the Kyoto Encyclopedia of Genes and Genomes pathways encoded in the gut microbiota. Serum metabolites were analyzed using nuclear magnetic resonance-based metabolomics coupled with multivariate statistical analyses. Hepatic gene expression profiles were analyzed *via* DNA microarray and quantitative polymerase chain reaction.

**Results:**

CDAHFD60 feeding induced hepatic steatosis, and in combination with bacterial administration, it further aggravated NAFLD pathology, thereby increasing fibrosis. Gene expression analysis of liver samples revealed that genes involved in NAFLD pathology were perturbed, and the two bacteria induced distinct expression profiles. This might be due to quantitative and qualitative differences in the influx of bacterial products in the gut because the serum endotoxin levels, compositions of the gut microbiota, and serum metabolite profiles induced by the ingested *P. intermedia* and *P. gingivalis* were different.

**Conclusions:**

Swallowed periodontopathic bacteria aggravate NAFLD pathology, likely due to dysregulation of gene expression by inducing gut dysbiosis and subsequent influx of gut bacteria and/or bacterial products.

## Introduction

Nonalcoholic fatty liver disease (NAFLD), a hepatic manifestation of metabolic syndrome and obesity, affects 20%–30% of the general Western population, and the associated morbidity is continuously increasing (1). NAFLD constitutes a spectrum ranging from simple steatosis and nonalcoholic steatohepatitis (NASH) to fibrosis, cirrhosis, and hepatocellular carcinoma. NAFLD is now considered to be a multifactorial disease involving multiple intracellular signaling pathways (2), dietary factors (3, 4), gut barrier dysfunction, endoplasmic reticulum (ER) stress, microbiota, and genetic factors (5).

Periodontal disease is a chronic inflammatory disease caused by a complex interaction between oral pathobionts and host defense mechanisms (6) that affects tooth-supporting structures, leading to tooth loss if left untreated. Accumulating evidence strongly suggests that periodontal disease not only destroys the periodontium, but also increases the risk of various non-oral diseases including metabolic disorders such as NAFLD (7). Epidemiological studies have demonstrated a significant association between clinical and/or microbial periodontal parameters and NAFLD (8–10). In addition, animal studies employing infection with periodontopathic bacteria such as *Porphyromonas gingivalis*, a representative periodontopathic bacterium with various unique virulence factors (11), or ligature-induced periodontitis have shown that these experimental conditions aggravate the clinical manifestations of NAFLD (10, 12, 13).

The possible mechanisms by which periodontal disease exacerbates NAFLD conditions have been considered to include endotoxemia and diffusion of inflammatory mediators from the periodontal tissue to the systemic circulation. These notions are based on studies showing that periodontal bacterial DNA is present in the various locations, such as the atheroma tissue, and that elevated serum levels of proinflammatory cytokines and high-sensitivity C-reactive protein are elevated in periodontitis patients (7). Although dental procedures, especially scaling and root planing, facilitate the entry of bacteria residing in periodontal pockets into the bloodstream (14), spontaneous bacteremia in patients with periodontal disease is rarely seen without such intervention (15). In addition, oral bacteria other than periodontopathic bacteria and enterobacteria can be detected in the vascular lesions of patients with cardiovascular disease and periodontitis, with a prevalence notably higher than that of periodontopathic bacteria (16). Therefore, there may be another causal mechanism responsible for the link between periodontal disease and NAFLD.

Recent observations strongly suggest that the gut microbiota plays a substantial role in the development of NAFLD in humans (17–19), as well as in animal models (20, 21). In this regard, we have demonstrated that orally administered *P. gingivalis* induces alterations in the composition of gut microbiota (22–25). The dysbiosis induced by periodontopathic bacteria is closely associated with decreased expression of tight junction proteins, endotoxemia, and the inflammatory phenotype of various tissues, including liver tissue and adipose tissue. These inflammatory changes are also associated with NAFLD, suggesting that changes in the gut microbiota caused by orally administered *P. gingivalis* may be involved in the pathogenesis and progression of NAFLD. Although *P. gingivalis* is the most well-known and extensively investigated periodontopathic bacterium, the effect of other bacteria, such *Prevotella intermedia* [which was reported to have minor effects on gut microbiota in a collagen-induced arthritis model (24)], are not known. Therefore, the present study was designed to investigate the underlying causal mechanisms through which periodontal disease increases the risk of NAFLD leveraging the oral–gut connection.

## Materials and Methods

### Ethics Statement

This study was approved by the Institutional Animal Care and Use Committee of Niigata University (permit number; SA00328). All experiments were performed in accordance with the Regulations and Guidelines on Scientific and Ethical Care and Use of Laboratory Animals of the Science Council of Japan, enforced on June 1, 2006.

All authors had access to the study data and had reviewed and approved the final manuscript.

### Bacterial Cultures

*P. gingivalis* strain W83 and *P. intermedia* ATCC25611 maintained in our laboratory were cultured in modified Gifu anaerobic medium broth (Nissui, Tokyo, Japan). *Veillonella rogosae* JCM 15642^T^ and *Actinomyces naeslundii* ATCC19039 obtained from Dr. Mashima at Aichi-Gakuin University, Nagoya Japan and maintained in our laboratory, respectively were cultured in brain–heart infusion broth. *P. gingivalis* and *P. intermedia* were used as periodontopathic bacteria whereas *A. naeslundii* and *V. rogosae* were used as commensal controls.

### Dietary Treatment and Bacterial Administration

**Figure 1:**
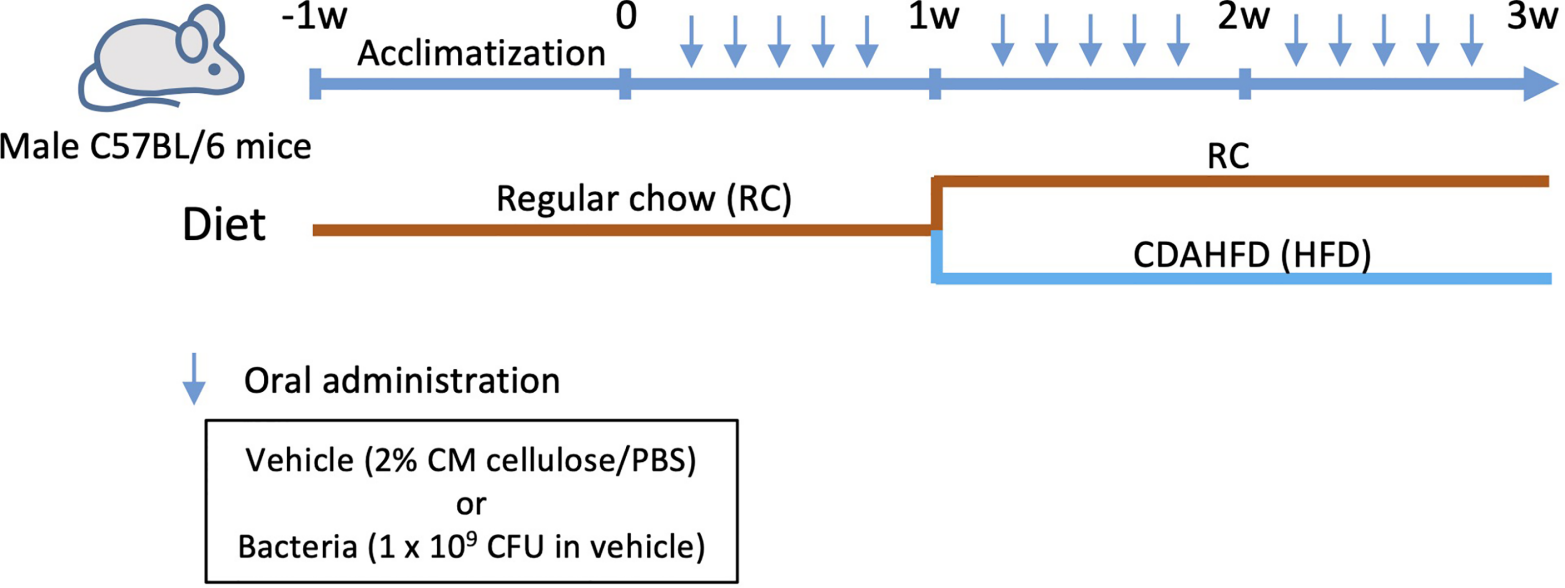
Flowchart depicting the experimental workflow.

Six-week-old male C57BL/6N mice were obtained from Japan SLC (Shizuoka, Japan). After acclimatization under specific pathogen-free conditions and feeding regular chow and sterile water for 1 week, the mice were orally administered with any one of the following: vehicle (PBS with 2% carboxymethyl cellulose; Sigma-Aldrich, St. Louis, MO), or a total of 1 × 10^9^ colony-forming units of each bacterial species suspended in vehicle through a feeding needle five times a week for 3 weeks. The number of administered bacteria was determined by considering the body weight and the number of bacteria in the saliva of periodontitis patients (26–28). At 1 week after the commencement of infection, the diet was changed to CDAHFD60 (#A06071302, Research Diets Inc., New Brunswick, NJ), except for the negative control group that was only administered the vehicle only until the end of the experiment. To analyze the effect of bacteria alone, an experimental group without diet change was also set (**Figure 1**).

### Liver Histology and Biochemical Analyses

Part of the left and medial lobes were fixed in neutral-buffered formalin. After deparaffinization and rehydration, paraffin-embedded sections (5 μm in thickness) were stained with hematoxylin and eosin (H&E) or were subjected to Masson’s trichrome staining to visualize collagen fibrils.

Liver triglycerides were determined using a Triglyceride Quantification Colorimetric kit (BioVision Inc., Milpitas, CA, USA). Triglyceride levels were expressed as the concentration of triglycerides divided by the total protein concentration. The hepatic hydroxyproline level was photometrically measured using a commercially available kit (Quickzyme Bioscience, Leiden, Netherlands), according to the manufacturer’s instructions.

### Sequencing of 16S rRNA and Analysis of the Gut Microbiota

Feces were collected and stored at −80°C until ready for use. Fecal DNA extraction was performed as described previously (29, 30). DNA was extracted from fecal samples by using lysozyme (Wako Pure Chemical Industries, Osaka, Japan), achromopeptidase (Wako Pure Chemical Industries), and proteinase K (Merck & Co., Inc., Kenilworth, NJ).

Bacterial DNA from fecal samples was amplified by PCR, as described previously (25). Primers (515F and 806R) with the adaptor sequencing for the Illumina MiSeq platform (Illumina, San Diego, CA, USA) were used. PCR amplification using Ex Taq Hot Start Version (Takara Bio, Shiga, Japan) was performed for 25 cycles. Amplicons were purified with AMPure XP (Beckman Coulter, Brea, CA, USA) and sequenced using the Illumina MiSeq platform.

### Metagenomic Sequencing and Analysis

Metagenome shotgun libraries (insert size of 500 bp) were prepared using the TruSeq Nano DNA kit (Illumina) and sequenced on the Illumina NovaSeq platform. After quality filtering, reads mapped to the reference human genome (HG19), and the phiX bacteriophage genome were removed. For each individual, filter-passed NovaSeq reads were assembled using MEGAHIT (v1.2.4). Contigs with a length <500 bp were removed. Gene prediction was performed using the Prodigal software (31). The nucleotide sequences of the predicted genes were clustered using CD-HIT-est v.4.6, with ≥95% sequence identity and ≥90% coverage. The clustered gene sequences were translated into proteins. Functional annotation of the predicted proteins against the KEGG database (release 63) was performed using DIAMOND v0.8, with an E-value of 1e−5. A total of 1 million high-quality reads were mapped onto the nonredundant gene set using bowtie2 to quantify the functional composition of each sample.

### Serum Markers

Serum levels of ALT and AST were analyzed using commercially available kits (BioVision, Inc., Milpitas, CA, USA), according to the manufacturer’s instructions.

### Endotoxin Assay

Serum endotoxin levels were determined using a Limulus Amoebocyte Lysate Test (Toxicolor™ LS50M, Seikagaku Co., Tokyo, Japan), according to the manufacturer’s instructions. Serum samples were 1:4 diluted for the assay. Optical densities were measured using an enzyme-linked immunosorbent assay (ELISA) plate reader (Emax Plus, Molecular Devices, San Jose, CA, USA) at 545 nm.

### DNA Microarray Analysis

Total RNA from the tissue samples was extracted using the TRIzol reagent (Molecular Research Center) 24 h after the final bacterial or sham administration, and quantified using a NanoDrop 2000 (Thermo Scientific, Wilmington, DE, USA). The total RNA was labeled and then hybridized to an Agilent SurePrint G3 Mouse Gene Expression 8 x 60K mRNA microarray chip (Agilent Technologies). All microarray experiments were conducted in Macrogen, Japan (Kyoto, Japan).

Microarray results were extracted using the Agilent Feature Extraction software v11.0 (Agilent Technologies). Hierarchical cluster analysis was performed using complete linkage and Euclidean distance as a measure of similarity. Gene enrichment and functional annotation analysis for the significant probe list was performed using the GO resource (www.geneontology.org/). All data analyses and visualization of differentially expressed genes were conducted using R 3.3.2 (www.r-project.org).

### Gene Set Enrichment Analysis

Hierarchical cluster analysis was carried out using Ward’s linkage (ward.D2) with Euclidean distance (32), and transcriptomic data were clustered into 21 groups. Among the 21 clusters, we selected seven characteristic clusters with up- or downregulated genes in *Pg* mice. Enrichment analyses were performed to obtain more information about the biological functions and pathways significantly enriched in up- and downregulated genes by focusing on the GO term biological process and KEGG pathways using the DAVID enrichment analysis system (33). P-values were corrected for multiple testing using the Benjamini–Hochberg method implemented in DAVID (34).

### Quantitative Analysis of Gene Expression in the Liver and Intestines

cDNA was synthesized using the Transcriptor Universal cDNA Master (Roche Molecular Systems, Pleasanton, CA, USA). Primers and probes for real-time PCR were purchased from Life Technologies (Waltham, MA, USA). Reactions were carried out in a LightCycler 96 System (Roche) using TaqMan Gene Expression Assays (Life Technologies) as described previously (24). The LightCycler 96 software (Roche) was used to analyze the standards and perform quantification. The relative quantity of each mRNA was normalized to that of glyceraldehyde-3-phosphate dehydrogenase mRNA.

### Quantitation of Serum and Liver Metabolites

Serum samples diluted to one-sixth of their original concentration in 100 mmol/L potassium phosphate buffer (in deuterium oxide containing 1 mmol/L sodium 2,2-dimethyl-2-silapentane-5-sulfonate, pH = 7) were quantified using an Nuclear Magnetic Resonance (NMR) spectrometer (Bruker AVANCE II 700, Bruker Biospin, Rheinstetten, Germany) as described previously (25, 35). Intact liver samples were placed in zirconia 4 mm diameter zirconia rotors and analyzed by ^1^H high-resolution magic angle spinning (hr-MAS) NMR spectroscopy at 500.132 MHz, with a spin rate of 4000 Hz (36). To annotate the signals detected in the ^1^H NMR spectra, two-dimensional *J*-resolved (*J*-res) NMR measurements (Bruker standard pulse program “jresgpprqf”) and hetero-nuclear single quantum coherence (HSQC) measurements (Bruker standard pulse program “hsqcetgpsisp2.2” were performed as described previously (37–39). The detected signals were annotated using the SpinCouple program (40) (http://dmar.riken.jp/spincouple/), and InterAnalysis program (41) (http://dmar.riken.jp/interanalysis/) based on HSQC and *J*-res cross peaks.

### Immunofluorescence Staining

Tissue sections above mentioned were deparaffinized with xylene, and rehydrated. This was followed by heat-induced antigen retrieval in BD Retrievagen A (pH 6.0, BD Biosciences, San Diego CA, USA). After blocking in fetal bovine serum (FBS), tissues were incubated with fluorescence-labeled anti-E-cadherin antibody (Alexa FluorTM 594 anti-mouse/human CD324, Biolegend, San Diego, CA, USA). After mounting with VECTASHIELD HardSet Mounting Medium with DAPI (Vector Laboratories, ABurlingame CA, USA), the stained sections were visualized by fluorescence microscopy (Biozero BZ-X710; Keyence Corporation, Osaka, Japan).

### Cell Culture

HepG2 cells were obtained from the American Type Culture Collection (Manassas, VA) and maintained in Dulbecco’s modified Eagle’s medium (DMEM) supplemented with 1% penicillin/streptomycin and 10% FBS in an atmosphere of 5% CO_2_ at 37°C. The cells were seeded in a 48-well plate at 3 × 10^5^ cells/well and cultivated for 24 h. Thereafter, the medium was changed to DMEM without FBS and the cells were cultured overnight. The cells were treated with a free fatty acid solution (final concentrations: 0.5 mM oleic acid and 0.25 mM palmitic acid) and fat-free bovine serum albumin for 24 h. The cells were then stimulated with LPS (1 μg/mL *P. gingivalis* or *P. intermedia* LPS, and 1 ng/mL *E. coli* LPS) for 4 h. Total RNA was extracted from the cells as described above and used for quantitative PCR.

### Bioinformatics and Statistical Analyses

Taxonomic assignments and estimation of relative abundance from sequencing data were performed using the analysis pipeline of the QIIME2 version 2020.6.0 (42). Amplicon sequence variants (ASVs) were inferred from the denoised reads using DADA2 (43) implemented in QIIME2. The ASV taxonomy was assigned based on a comparison with the SILVA version 138 (44). β-Diversity was calculated using weighted UniFrac distances based on the operational taxonomic unit distribution across samples and visualized by principal coordinate analysis (PCoA).

From the results of metagenomic analysis at TP2 and TP3, KEGG orthologies (KOs) with significant differences determined by the t-tests, which were carried out by using R, between two groups, except for RC mice, were extracted under the condition of *P* < 0.01. The KOs were mapped to the reference pathway in the KEGG database. Significant pathways were enriched with Fisher’s exact probability tests using the number of KOs mapped in each pathway. The p-values were adjusted by using the Benjamini–Hochberg method, which are used as q-values in the **Figure 6**. The quantified metabolome data were normalized using an autoscaling method and statistically analyzed using PCA.

Statistical analyses were performed using the GraphPad Prism version 9 (GraphPad Software, Inc., La Jolla, CA, USA) and R (version 4.0.4.). Randomization or blinding was not performed in the present study. All data are expressed as the mean ± standard error of the mean. Statistical analyses were performed using one-way analysis of variance with Tukey’s correction. Analysis of similarity was performed to identify differences in bacterial community compositions and PERMANOVA (PERmutational Multivariant Analysis Of Variance) was used for comparison of microbes between groups. Statistical significance was set at *P <*0.05.

## Results

### Oral Pathobionts, But Not Symbionts, Worsen NAFLD Pathology

**Figure 2:**
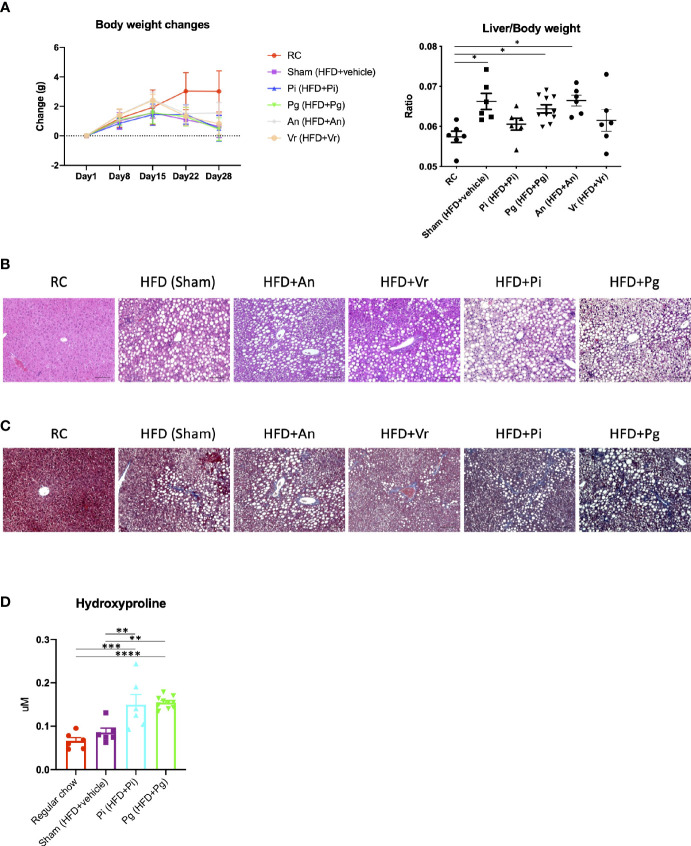
Bacterial administration exacerbates high fat diet-induced liver pathology. **(A)** Changes in body weight and liver/body weight ratios of mice in each group during the experimental period. *Pi* mice had a significantly lower body weight compared with RC mice at day 28 (n=6-10/group). **(B)** Hematoxylin and eosin staining of liver (scale bars, 100 μm). **(C)** Masson’s trichrome staining of the liver. **(D)** Hepatic contents of hydroxyproline (n=6-10/group). RC: C57BL/6N mice fed regular chow; Sham: Mice fed CDAHFD60 plus sham administration; *Pi*: Mice fed CDAHFD60 plus *P. intermedia* administration; *Pg*: Mice fed CDAHFD60 plus *P. gingivalis* administration, *An*: Mice fed CDAHFD60 plus *A naeslundii* administration, *Vr*: Mice fed CDAHFD60 plus *V. rogosae* administration. Data are expressed as the mean ± standard error of the mean (SEM). *P* values were calculated using one-way ANOVA with Tukey’s multiple comparisons test. *P < 0.05, **P < 0.005, ***P < 0.001, ****P < 0.0001.

Oral administration of bacteria did not affect body weight until commencement of the diet change, and no difference was observed among the vehicle-, *Actinomyces naeslundii-*, *Veillonella rogosae-*, *P. gingivalis*- and *P. intermedia*-administered mice (hereafter referred to as Sham, *An*, *Vr*, *Pg*, and *Pi* mice, respectively). After changing the diet from regular chow (RC) to choline-deficient, l-amino acid-defined, high-fat diet with 60 kcal% fat and 0.1% methionine (CDAHFD60) (CDAHFD60; HFD), a gain in body weight was suppressed in all experimental groups in comparison with RC-fed mice. In contrast, the liver-to-body weight ratio was significantly increased in Sham, *An*, and *Pg* mice compared with that in RC-fed mice (**Figure 2A**). HFD feeding promoted hepatic steatosis in all experimental groups, and the degree of steatosis was greater with *P. intermedia* administration, and it was further aggravated by *P. gingivalis* administration (**Figure 2B**). Similarly, the degree of fibrosis was progressively exacerbated with increasing bacterial burden (sham < *P. intermedia* < *P. gingivalis*) (**Figure 2C**). In contrast, no additional histological changes were observed with *A. naeslundii* and *V. rogosae* administration compared with those in Sham mice. Bacterial administration induced minimal histological changes in the liver of RC-fed mice except for *Pg* mice, in which slight steatosis was observed (**Supplementary Figure 1**). Therefore, further analyses were focused on *Pg* and *Pi* mice.

Under these conditions, the content of hepatic hydroxyproline increased with the increasing bacterial burden (**Figure 2D**). Although triglyceride content and aspartate transaminase (AST) and alanine transaminase (ALT) activities were significantly higher in HFD-fed groups, bacterial administration had no effect on them (**Supplementary Figure 2**).

### Effect of Bacterial Administration on Gut Barrier Function

**Figure 3:**
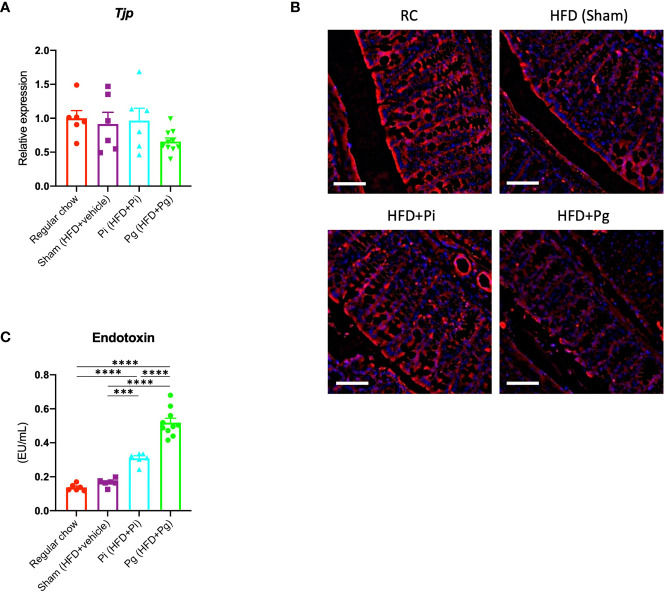
*P. gingivalis* administration induces gut barrier dysfunction. **(A)** Expression of *Tjp1* in the small intestines. cDNA was amplified with primers specific for *Tjp1* (n=6-10/group). The relative quantity of mRNA was normalized to that of glyceraldehyde-3-phosphate dehydrogenase mRNA. **(B)** Immunofluorescence analysis of E-cadherin in large intestines from each group. Red: E-cadherin, blue: DAPI, scale bars: 50 μm. **(C)** Serum endotoxin levels in the various groups (n=6-10/group). Data are expressed as the mean ± standard error of the mean (SEM). *P* values were calculated using one-way ANOVA with Tukey’s multiple comparisons test. ***P < 0.001, ****P < 0.0001.

Dysregulation of gut barrier function and subsequent endotoxemia are major contributors to NAFLD. Therefore, we analyzed whether the barrier function was altered in *Pg* mice, and if the barrier function was disorganized, whether endotoxemia was induced. As shown in **Figure 3A**, the expression of *Tjp1*, encoding tight junction protein, tended to be lower in *Pg* mice compared with other groups. Moreover, a decrease in the expression of E-cadherin was observed in the colon of *Pg* mice (**Figure 3B**). Additionally, the serum endotoxin level was increased with the increasing bacterial burden. The level was significantly higher in *Pi* mice compared with that in RC-fed and Sham mice, and it was further elevated in *Pg* mice (**Figure 3C**).

### Administration of Oral Bacteria Affects the Gut Microbial Composition and the Expression Profile of Genes in the Intestine

**Figure 4:**
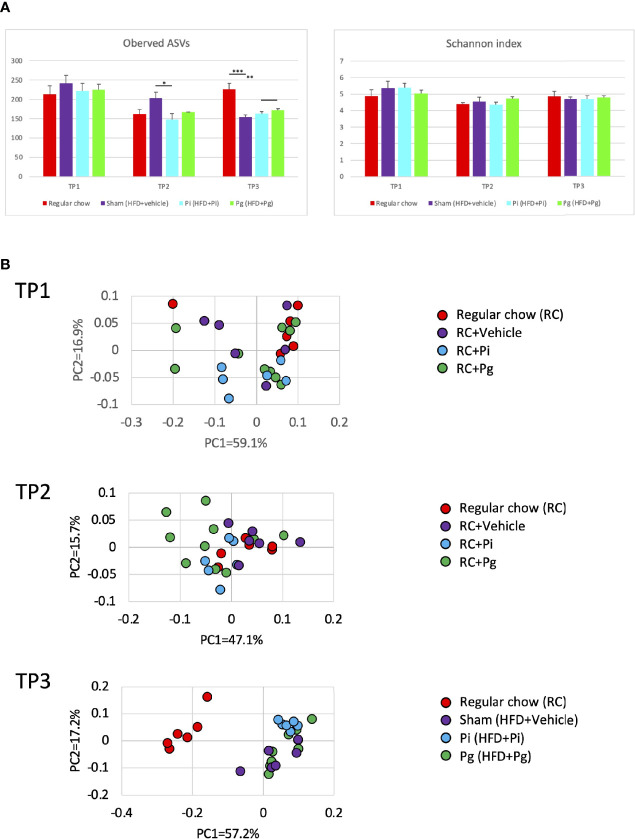
Effect of CDAHFD60 feeding and subsequent bacterial administration on the gut microbiota composition (n=6-10/group). Fecal samples from mice in various treatment groups were subjected to 16S rRNA sequencing. **(A)** Alpha diversity of each experimental group at different time points. ASV, Amplicon sequence variant. **(B)** Principal coordinate analysis score plot of the gut microbiota profiles of each experimental group at different time points using weighted UniFrac distance. Data are expressed as the mean ± standard error of the mean (SEM). *P* values were calculated using one-way ANOVA with Tukey’s multiple comparisons test. *P < 0.05, **P < 0.005, ***P < 0.001.

The global differences among experimental groups were evident from baseline (time point 1; TP1) to TP2, after 1 week of bacterial administration, as well as after additional HFD-feeding (TP3), according to the analyses of α- (**Figure 4A**) and β-diversities (**Figure 4B**).

**Figure 5:**
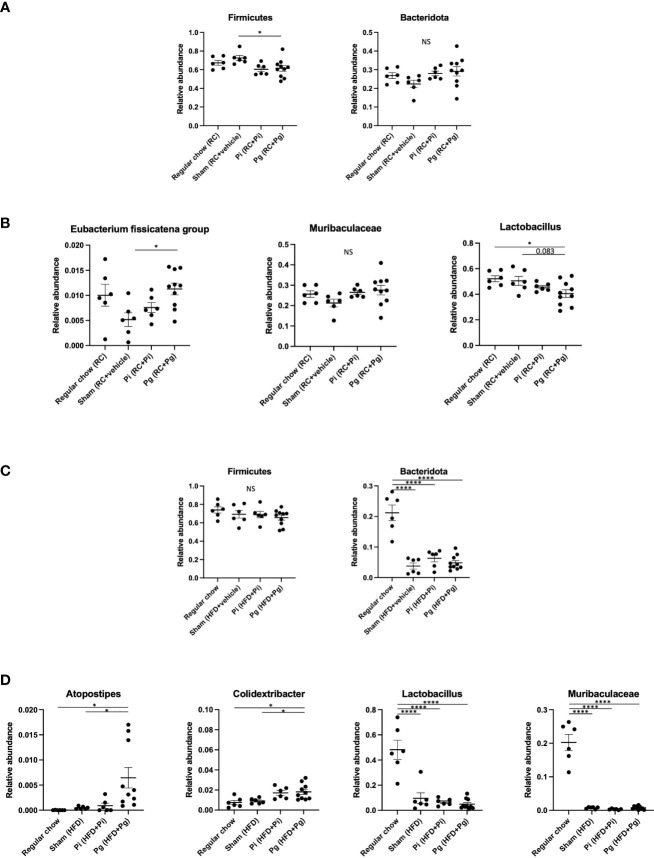
Effect of bacterial administration (TP2) and additional diet change (TP3) on the gut microbiota composition (n=6-10/group). Fecal samples from mice that received the various treatments were subjected to 16S rRNA sequencing. **(A)** Relative abundance of phyla Firmicutes and Bacteridota at TP2. **(B)** Relative abundances of characteristic genera in each experimental group at TP2. **(C)** Relative abundance of phyla Firmicutes and Bacteridota at TP3. **(D)** Relative abundances of characteristic genera in each experimental group at TP3. Data are expressed as the mean ± standard error of the mean (SEM). *P* values were calculated using one-way ANOVA with Tukey’s multiple comparisons test. *P < 0.05, ****P < 0.0001. NS, not significant.

At TP2, the proportion of the phylum Firmicutes was significantly lower in *Pg* mice than that in Sham mice (**Figure 5A**). However, the proportion of phylum Bacteridota (previously known as Bacteroidetes) did not differ among the experimental groups. At the genus level, the proportion of *Eubacterium fissicatena* group was significantly higher in *Pg* mice than in Sham mice. In contrast, the proportion of *Lactobacillus* was significantly lower and tended to be lower in *Pg* mice compared to RC-fed and Sham mice, respectively (**Figure 5B**).

HFD feeding had a strong effect on the gut microbiota composition. Despite the considerable effect of diet, bacterial administration induced a substantial change in the gut microbiota composition. Although no difference was observed in the phylum Firmicutes, HFD feeding significantly decreased the proportion of the phylum Bacteridota (**Figure 5C**). At the genus level, significantly elevated proportions of *Atopostipes* and *Colidextribacter* were observed in *Pg* mice compared with those in RC and Sham mice. The proportions of *Lactobacillus* and *Muribaculaceae* were significantly lower in HFD-fed mice and there was no effect of bacterial administration (**Figure 5D**). Although HFD feeding increased the abundance of many genera, there were some notable patterns of relative abundance among the different experimental groups. While *P. intermedia* administration further increased the relative abundance of some genera, it suppressed the effect of HFD feeding in some genera (**Supplementary Figure 3**).

Thus, hepatic pathology observed in *Pg* mice appeared to be directly related to gut dysbiosis. Consistent with these findings, the expression profile of genes in the intestine showed distinct patterns when compared between any two experimental groups (**Supplementary Figure 4**), suggesting that diet as well as the administered bacteria have a characteristic effect on gene expression, possibly through changes in the gut microbiota composition.

### Effect of Oral Pathobiont Administration on Metabolic Pathways in the Gut

**Figure 6:**
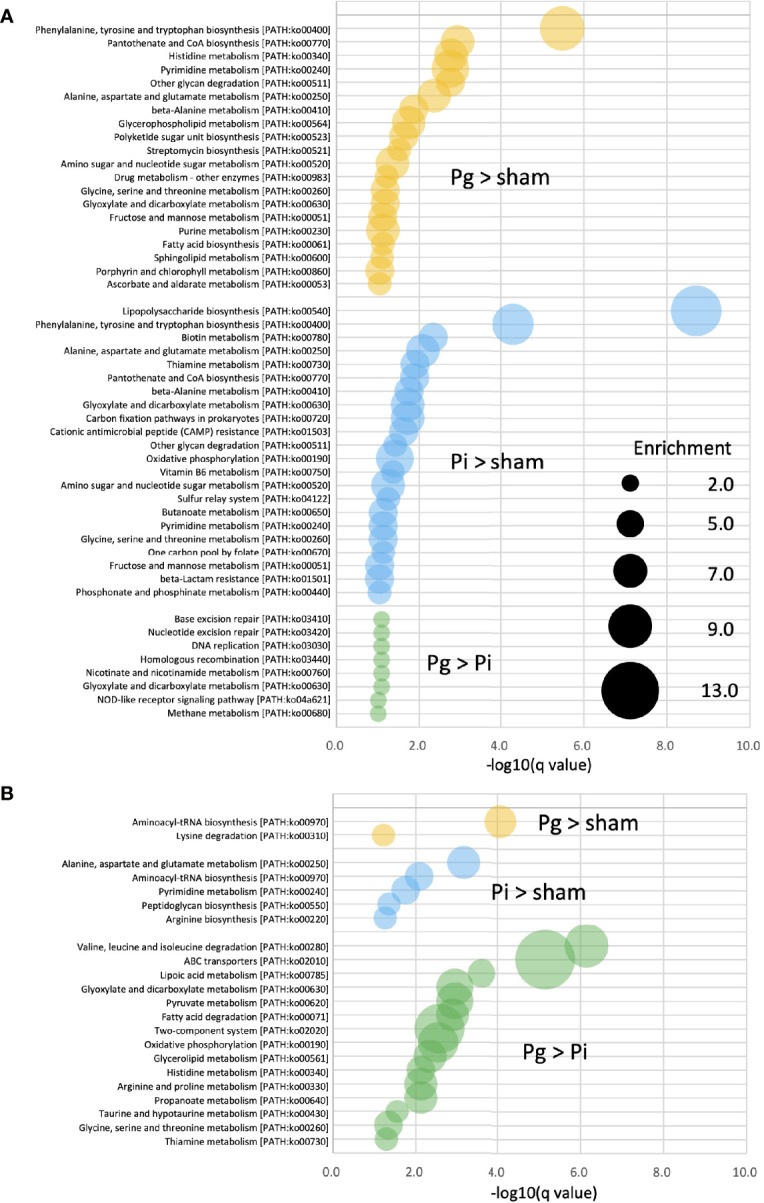
KEGG biomarkers from the metagenomic analysis of gut microbiota. Pathway analysis of differentially enriched genes between two groups. Enriched KEGG pathways (q < 0.1) at TP2 **(A)** and TP3 **(B)** are shown. The bubble size indicates the number of KOs enriched in each pathway. Expressions containing inequality signs (e.g., “*Pg* > Sham”) indicate that the results of enrichment analysis with Kos, in which the abundant of KOs for the left category (*Pg* mice) are significantly more abundant than those in the right category (Sham mice).

Enrichment analysis was applied to the relative abundance of Kyoto Encyclopedia of Genes and Genomes (KEGG) pathways *via* metagenomic analysis to study the function of gut microbiota in different groups at different time points. At TP2, in *Pg* and *Pi* mice, the enriched KEGG pathways overlapped with elevated amino acid metabolism, particularly, with respect to phenylalanine, tyrosine, and tryptophan biosynthesis. In *Pi* mice, the lipopolysaccharide biosynthesis pathway was also notably overrepresented. A total of eight enriched KEGG pathways, including the NOD-like receptor signaling pathway, were observed in *Pg* mice relative to the pathways observed in *Pi* mice (**Figure 6A**). Although the effect of bacterial administration on the gut metabolic pathways was diminished after HFD feeding (the number of pathways enriched at TP2 decreased compared to that at TP2), the difference between *Pg* mice and *Pi* mice was even more pronounced. Similar to TP2, amino acid metabolic pathways were the characteristic pathways when the two groups were compared. In addition, the pathways for fatty acid degradation and the two-component system were enriched in *Pg* mice relative to those in *Pi* mice (**Figure 6B**).

### Metabolic Profiles Are Affected by Bacterial Administration

**Figure 7:**
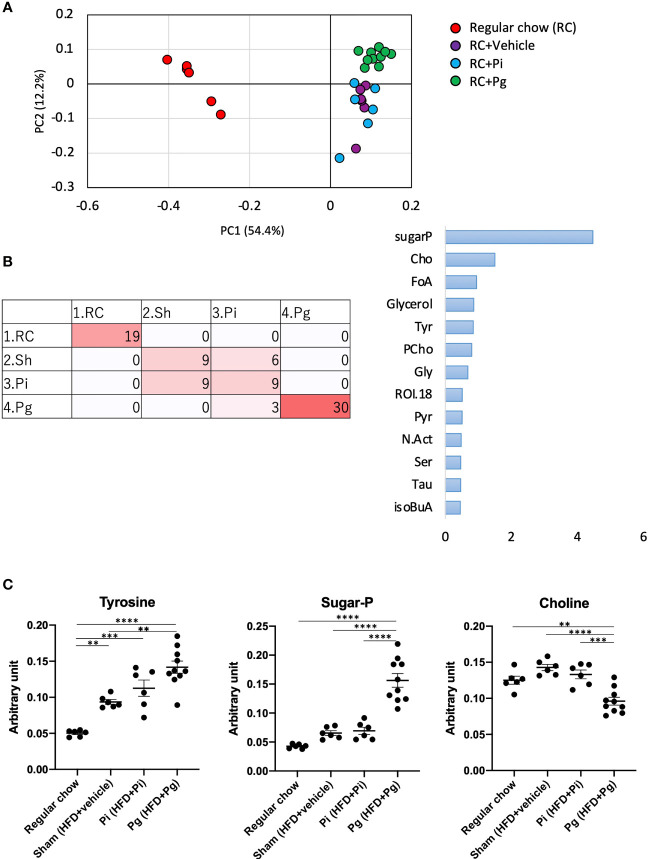
Effect of CDAHFD60 feeding and subsequent bacterial administration on serum metabolites. **(A)** PCA of serum metabolites in each group. **(B)** Machine learning (random forest) classification of each group. Left: confusion matrix (RC, regular chow; Sham, HFD + vehicle; Pi, HFD + Pi; Pg, HFD + Pg). Right: important variables (metabolites) contributing to four classifications. Tyr, tyrosine; CHO, choline; FoA, formate; Gly, glycine; CiA, citrate; GPC, glycerophosphocholine; Ac, acetate; ROI, region of interest. **(C)** Compounds that differed in abundance among groups (n=6-10/group). Data are expressed as the mean ± standard error of the mean (SEM). *P* values were calculated using one-way ANOVA with Tukey’s multiple comparisons test. **P < 0.005, ***P < 0.001, ****P < 0.0001.

We further analyzed liver tissue and serum samples to assess whether the murine metabolome was perturbed by bacterial administration, in addition to being affected by diet. We performed an untargeted analysis of all the data acquired to examine a wider pool of metabolites. Principal component analysis (PCA) was conducted using nuclear magnetic resonance (NMR)-derived data to obtain an overview of the differences among the groups. This analysis not only differentiated HFD-fed mice from RC-fed mice, but also highlighted the effect of *P. gingivalis* administration among HFD-fed mice in the liver tissue and serum metabolites (**Supplementary Figure 5A** and **Figure 7A**), which was consistent with the histological findings. Furthermore, the machine-learning model allowed a discrete classification within the four groups in both tissue and serum metabolites; maltose, choline, a fatty acid (FA.CH3.n.3), choline metabolite (CHONCH3), and methionine were among the characteristic metabolites in the tissue (**Supplementary Figure 5B**), whereas metabolites such as ROI.38 [which has been suggested to be a sugar-phosphate (sugar-P)], tyrosine, and choline were revealed to be important in the serum (**Figure 7B**). The tissue profile of short-chain fatty acids (SCFAs) showed no difference among the groups (**Supplementary Figure 6A**). In contrast, the levels of acetate and citrate were significantly higher in HFD-fed mice compared with those in RC-fed mice and tended to increase with an increasing bacterial burden, with the highest level in the serum of *Pg* mice. However, there was no difference in the level of the other SCFAs (**Supplementary Figure 7A**). With respect to the amino acid levels in the tissue, the level of lysine was significantly decreased in HFD-fed mice, with a further decrease in *Pg* mice compared with Sham mice. A similar trend was observed for threonine (**Supplementary Figure 6B**). The level of ChoNCH3, a choline metabolite, showed a pattern similar to that of choline (**Supplementary Figure 5C**). The levels of lipid metabolites were increased in HFD-fed mice; however, bacterial administration had a minimal effect on these levels (**Supplementary Figure 5C**). In the sera, HFD feeding significantly elevated the serum levels of the annotated signals, except for isoleucine, glutamine, and phenylalanine (**Supplementary Figure 7B**). Notably, tyrosine levels were significantly elevated in *Pg* mice compared with those in Sham mice. Similarly, sugar-P levels were significantly higher in *Pg* mice compared with those in the other groups. The choline levels were drastically lower in *Pg* mice compared with those in the other groups (**Figure 7C**). The levels of other annotated molecules were also elevated in HFD-fed mice, except those of allantoin, which increased with increasing nutrition and bacterial burden, and there was a significant difference in these levels between RC-fed and *Pg* mice (**Supplementary Figure 7C**).

### Oral Administration of Periodontopathic Bacteria Modulates the Hepatic Expression of Genes Implicated in NAFLD Pathology

Although the effect of HFD feeding on the liver was robust, it was evident that the administration of both bacteria induced additional and substantial changes in the expression profile of genes. Moreover, the expression profile of genes in the liver was clearly distinct between *Pi* and *Pg* mice (**Supplementary Figure 8**). Therefore, we evaluated the expression of genes in the liver using functional enrichment analysis.

Based on hierarchical clustering, we extracted seven clusters in which genes in clusters 1–3 were downregulated, whereas those in clusters 4–6 were upregulated in *Pg* mice compared with the other groups. Cluster 7 included genes, the expression levels of which were higher in *Pg* mice than those in RC-fed mice, but lower compared with those in Sham and *Pi* mice (**Supplementary Figure 9**). The genes in these clusters are listed in **Supplementary Table 1**.

In cluster 1, significantly downregulated genes were mostly annotated as those involved in the biosynthesis and metabolic processes of lipids, organic acids, oxoacids, steroids, and fatty acids. No gene ontology (GO) terms were enriched in clusters 2 and 3. In cluster 4, genes involved in the cell cycle, cell death, nuclear division, DNA replication, responses to oxidative and ER stress, and regulation of intrinsic apoptosis were upregulated. In cluster 5, genes involved in the inflammatory response, such as in the regulation of leukocyte migration, response to lipopolysaccharide (LPS), cellular response to inflammatory cytokines, and nuclear factor-κB (NF-κB) pathways, were significantly enriched. In cluster 7, genes involved in the biological processes for circadian regulation of gene expression were significantly enriched (**Supplementary Table 2**).

**Figure 8:**
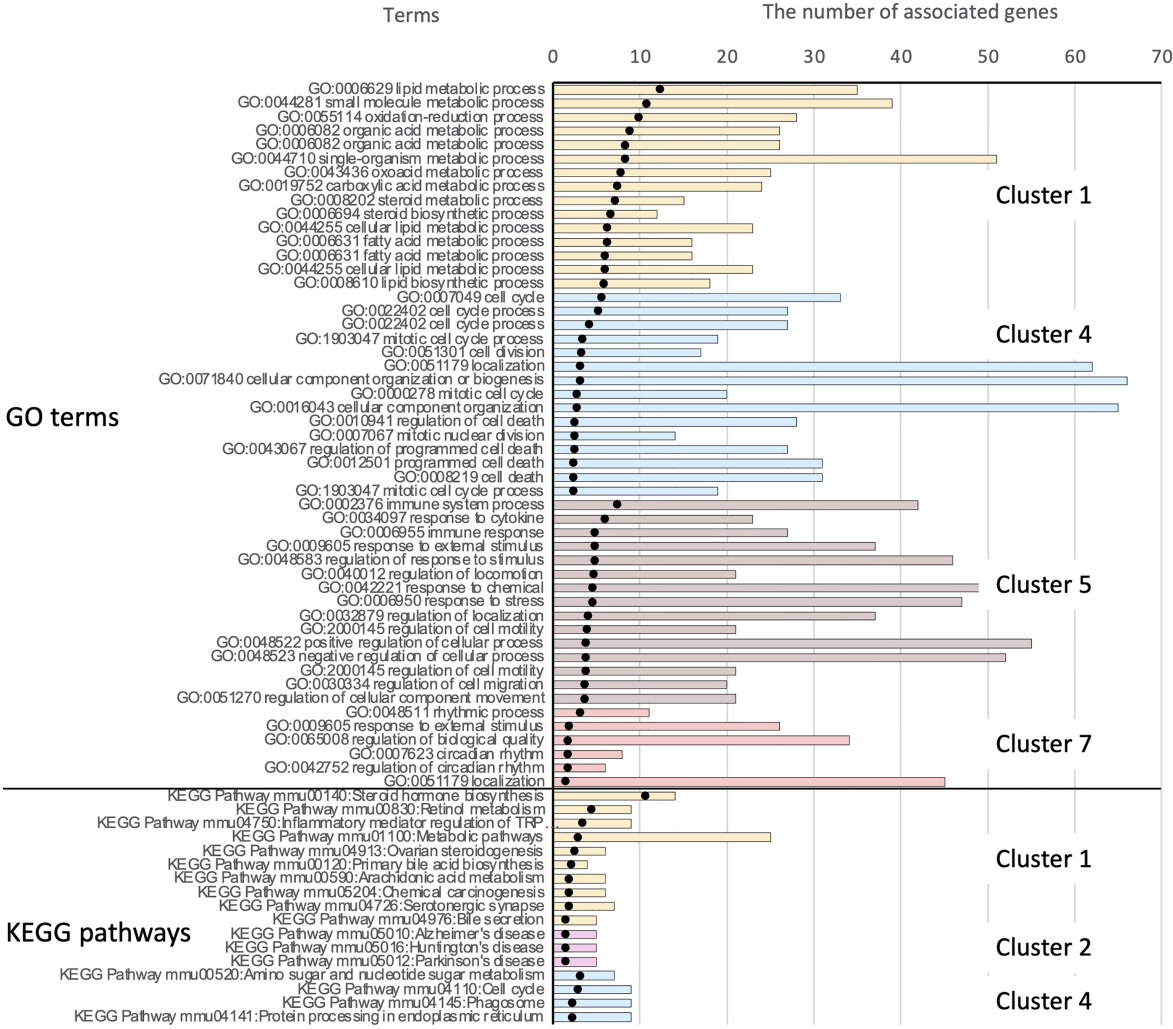
Results of functional enrichment analysis. Owing to the large number of significant GO terms (corrected p < 0.05), only the top 15 significant terms from each cluster are shown. For results of KEGG pathway analysis, all significant pathways are presented. Black dots indicate the –log10 of Benjamini-Hochberg-corrected p-values. The top 51 bars show the results of GO enrichment, and the bottom 17 bars show enriched KEGG pathways. Bars showing the associated clusters are indicated.

The significantly enriched KEGG pathways in clusters 1, 2, and 4 are shown in **Supplementary Table 3**. These included various pathways for steroid hormones, retinol, primary bile acids, arachidonic acid, amino sugars, and nucleotide sugars. Some metabolic pathways were consistent with the enriched Gene Ontology (GO) terms in cluster 1. The top 15 significant GO terms and the complete list of enriched KEGG pathways in each cluster are shown in **Figure 8**.

Progression from simple steatosis to steatohepatitis, fibrosis, and hepatocellular carcinoma is believed to be mediated by multiple parallel factors, including inflammation, ER stress, lipotoxicity, and altered circadian rhythms. The GO terms and KEGG pathways implicated in these events were found to be significantly enriched in the clusters (**Supplementary Tables 2, 3**). To confirm the data of DNA microarray analysis, quantitative real-time PCR was conducted for representative genes involved in the pathological mechanisms of NAFLD. Genes involved in the inflammatory responses were upregulated by HFD feeding, irrespective of the bacterial administration. The expression of *Tsc22d3*, which mediates the anti-inflammatory response, was only elevated in *Pg* mice, reflecting a weaker elevation of inflammatory genes in this group (**Supplementary Figure 10A**).

Fibrosis- and ER stress-related genes were enriched and classified in cluster 4. Whereas *Col1a1* and *Timp1* were upregulated by HFD feeding, *Ctgf* was upregulated specifically by *P. gingivalis* administration (**Supplementary Figure 10B**). ER stress-related genes *Chop*/*Ddit3* and *Ddit4* were also elevated by *P. gingivalis*. Consistent with these findings, *Fgf21* and *Trib3*, both of which are induced by ER stress and have been reported to be elevated in NAFLD, were significantly upregulated in *Pg* mice, but not in *Pi* mice (**Supplementary Figure 10C**).

The GO terms associated with the cell cycle process, which are potentially implicated in carcinogenesis and end-stage NAFLD, were also enriched in cluster 4. The expression of *Hnf6* and *Hhex* was upregulated in *Pi* mice, but not in *Pg* mice (**Supplementary Figure 10D**). The expression of *Hnf6* in hepatocellular carcinoma (HCC) cells is negatively associated with their malignancy. Delivery of *Hhex* into a hepatoma cell line has been reported to decrease the expression of several proto-oncogenes and to increases the expression of some tumor suppressor genes (45).

In cluster 7, GO terms associated with controlling the circadian rhythm were enriched. Fluctuations in the circadian rhythm affect metabolism and alter the expression of liver clock genes in NAFLD pathology. In this connection, the clock gene, *Per1*, was significantly elevated in *Pg* mice. *Pg* and *Pi* mice demonstrated contrasting expression patterns of *Bmal1* and *Dbp*. (**Supplementary Figure 10E**).

These distinctive expression profiles of hepatic genes in RC-fed, *Pi*, and *Pg* mice may not be due to the direct effect of administered bacteria, but rather due to differences in the gut microbiota compositions triggered bacterial administration. In support of this assumption, the gene expression of ER stress-related genes in HepG2 cells stimulated with LPS from *P. intermedia*, *P. gingivalis*, and *E. coli* was different from that in the liver of each experimental group (**Supplementary Figure 11**).

## Discussion

Recent indisputable evidence suggests that gut dysbiosis is a driver of NAFLD progression. An association between gut dysbiosis and NAFLD pathology has been described in the context of increased gut permeability, exposure of the liver to bacteria and bacterial products, and altered metabolites produced by the gut microbiota (46). Oral dysbiosis is a characteristic feature of periodontitis patients, and these patients continuously and unconsciously swallow pathobionts present in the saliva. Therefore, the concept that swallowed pathogenic oral microbiota induces gut dysbiosis is likely to be another possible causal mechanism linking periodontitis and NAFLD. In fact, in this study, the oral pathobionts *P. intermedia* and *P. gingivalis*, but not the oral symbionts *A. naeslundii* and *V. rogosae*, worsened HFD-induced NAFLD, with *P. gingivalis* showing higher pathogenicity. These pathological changes coincided with the reduction in the gut barrier function and serum levels of endotoxin, which is a gut dysbiosis-related risk factor for NAFLD.

Consistent with the results of our previous study and those of others, gavage of oral pathobionts induced changes in the gut microbiota composition (**Figure 4**). Despite the pronounced effect of diet change on the gut microbiota composition, the effect of oral pathobionts remained obvious. Furthermore, a significant difference in the microbiota composition was observed between *Pi* and *Pg* mice. Because the effect of *P. gingivalis* was not distinctive compared to that of *P. intermedia* at TP2, ingestion for a longer time and consumption of HFD may synergistically affect the gut microbiota composition.

*P. gingivalis*, but not *P. intermedia*, induced a significant reduction in the proportion of bacteria belonging to the phylum Firmicutes (**Figure 5**). Among the bacteria in the phylum Firmicutes, the proportion of *Lactobacillus* was significantly decreased in *Pg* mice, supporting the findings of the protective role of *Lactobacillus* against NAFLD (47, 48). In addition, significant changes in the *Eubacterium fissicatena* group were observed at TP2. Although some species of *Eubacterium* have been reported to be contribute to NAFLD pathogenesis (49), the role of these bacteria remains to be determined.

Additionally, the fundamental effect of *P. gingivalis* on the gut microbiota was evident after the change in diet (TP3 in **Figure 4B**). Although the role of these bacteria in liver pathology has not been clarified, *Colidextribacter* has recently been reported to be positively correlated with serum levels of oxidative markers (50) and hyperlipidemia-related parameters (51). In addition, functional enrichment analysis of the expression profiles of genes in the liver demonstrated that the genes involved in the response to oxidative stress were enriched (Cluster 4) in *Pg* mice.

Metagenomics analysis demonstrated the accumulation of genes implicated in the different KEGG pathways characteristic of each experimental group, suggesting a functional alteration of the gut microbiota by different pathobionts (**Figure 6**). At TP2, phenylalanine, tyrosine, and tryptophan biosynthesis pathways (ko00400) and alanine, aspartate, and glutamate metabolism pathways (ko00250) were significantly enriched in *Pg* and *Pi* mice compared with Sham mice. Alanine, aspartate, and glutamate metabolism pathways have recently received attention as risk factors in the development of NAFLD. Dysregulated glutamine metabolism has also been implicated in NAFLD pathology (52). Despite the significant effect of HFD on gut microbiota, the effect of oral pathobionts was still obvious, given the enriched amino acid metabolism at TP3. In addition, fatty acid metabolism and the two-component system were enriched in *Pg* mice, as compared with *Pi* mice. The former (ko00071) is related to increased levels of L-palmitoyl-carnitine, which has been reported to be associated with ischemic heart disease (53), and the latter (ko02020) is known to regulate various virulence genes (54), suggesting a pathogenic role of oral pathobionts against the gut microbiota.

A change in the gut microbiota composition can affect NAFLD pathology *via* various pathways and involved metabolites that are the products of gut microbial metabolism. Such bioactive metabolites are absorbed from the intestinal tract into the systemic circulation *via* the portal vein; therefore, serum metabolomic data can partially reflect the level of gut microbial metabolites. Although a significant effect of diet on the serum metabolomic profile was anticipated at TP3, it is noteworthy that the profile of *Pg* mice was clearly distinct from that of RC-fed and *Pi* mice (**Figure 7A**). Among several metabolites that were found to be differentially abundant in each group, tyrosine was of particular interest. The serum levels of tyrosine were significantly elevated in HFD-fed mice and were further increased in *Pg* mice. Recently, serum levels of amino acids have been shown to be associated with metabolic diseases. Aromatic amino acids have been shown to be associated with the risk of developing not only diabetes (55) and cardiovascular disease (56) but also NAFLD (57, 58). Another molecule of note is choline. Choline deficiency contributes to the development of fatty liver disease through multiple mechanisms, which are fundamental to the animal model used in this study. The level of choline, as a component of lard, was low in the HFD, not meeting the level necessary for optimal health (59). Therefore, the change from RC to the HFD induced NAFLD. Although the HFD was equally fed to Sham, *Pi*, and *Pg* mice, *P. gingivalis* administration further lowered the serum choline levels (**Figure 7C**). In addition to low dietary choline intake, the estrogen status, single nucleotide polymorphisms (60), and gut microbiota are important modulators of choline bioavailability (61), suggesting that the low level of choline in *Pg* mice was highly attributable to the change in gut microbiota. Recently, eight strains of bacteria in the human gut, mostly belonging to the order Clostridiales, were identified to produce trimethylamine from choline (62). This metabolic pathway is highly relevant, with respect to an increased risk of atherosclerosis *via* elevation of trimethylamine-N-oxide levels (63). However, the relative abundance of bacteria belonging to the order Clostridiales was not different among the experimental groups. Therefore, it is assumed that other bacteria may be associated with low levels of serum choline in *Pg* mice. In addition, sugar phosphate was characterized as a metabolite in *Pg* mice. Although the reason for this finding is not known, this may indicate cell damage because phosphorylated sugars are usually localized in cells.

Interplay between the gut microbiota, including the production of byproducts, is the primary mechanism underlying the pathogenesis of NAFLD. DNA microarray and subsequent qPCR analyses of the liver tissue provided insights into the pathological mechanisms induced by different periodontopathic bacteria. Altered expression of genes in the liver caused by administration of either *P. gingivalis*- or *P. intermedia* became evident as a driving force for a more severe disease phenotype by influencing various aspects of disease mechanisms (**Supplementary Figure 10**). Functional enrichment analysis revealed the activation of multiple pathological pathways in the liver by oral pathobionts, particularly *P. gingivalis*. These include genes associated with the NF-κB pathway, ER stress, circadian rhythm, fibrosis-, and tumorigenesis. Among these, the modulation of the expression of ER stress-related genes is of particular interest.

Recently, the importance of ER stress in various aspects of NAFLD has been highlighted (64). Excessive calorie intake and the resulting accumulation of lipids in hepatocytes evoke cellular stress pathways. This type of cellular stress originates from the accumulation of unfolded or misfolded proteins in the ER and usually triggers an adaptive response to resolve the ER stress, namely the unfolded protein response (UPR).

*Chop*/*Ddit3* is involved in the activation of NF-κB signaling (65) and the promotion of apoptosis in hepatocyte (66), mediating inflammation and fibrosis, and the progression from steatosis to NASH, respectively. Furthermore, another gene, *Trib3*, which has a deleterious effect on insulin signaling in hepatocytes under the control of Chop (67) was significantly upregulated by *P. gingivalis* administration. Similarly, the levels of *Ddit4*, another ER stress-related gene, the expression of which is also mediated through the Perk, IRE1α, and ATF6-dependent cascade (68), were elevated. Considering that the expression of both *Chop* and *Ddit4* in Sham mice was comparable with that in RC-fed mice, the effect of HFD feeding itself might have been well controlled by the UPR, but the additional effect of *P. gingivalis* administration was beyond the well-controlled UPR.

Another notable finding was the disruption of the molecular clock by bacterial administration (**Supplementary Table 3** and **Supplementary Figure 10E**). Factors affecting NAFLD pathology, such as liver metabolic pathways, bile acid synthesis, and immune/inflammatory processes, show circadian patterns driven by the biological clock. Therefore, disruption of the circadian clock leads to various diseases, including NAFLD (69). Although it has been demonstrated that diet-induced dysbiosis disturbs the balance between microbes and host circadian networks, which affects metabolism and obesity (70), orally administered bacteria further modulate the expression of clock genes with distinct patterns induced by different bacteria, suggesting another causal relationship between periodontitis and NAFLD.

The end-stage of NAFLD is HCC. Although histological changes and distinct expression of oncogenes were not observed, carcinogenesis-related genes were enriched in cluster 4 (upregulated in *Pg* mice compared with the other groups; **Supplementary Table 2**), in addition to cellular stress- and inflammation-related genes that are indirectly associated with carcinogenesis. The expression of tumor suppressor genes was significantly elevated in *Pi* mice (**Supplementary Figure 10D**). In addition, these results suggest that many of the differentially expressed genes in the liver of *Pg* mice were directly or indirectly involved in increased production of secondary bile acid, cytokine responses, and cellular responses to LPS, which are considered to be consequences of impaired gut barrier function.

In the present study, using a nutrient-deficient NAFLD model, orally administered periodontopathic bacteria (especially *P. gingivalis*) induced a change in the gut microbiota composition and serum metabolome, resulting in alteration of the liver transcriptome toward aggravation of the pathology in NAFLD. These results provide new insights into the mechanisms by which periodontitis contributes to NAFLD pathology.

Although our mouse model can be considered to be a reliable physiological model that is clinically relevant to the human disease, it does not fully reflect pathology in humans. For example, it lacks the development of significant obesity and glucose intolerance (71). Moreover, administration of a single species of periodontopathic bacteria does not completely replicate the condition of periodontitis patients, although the number of bacteria required to do so is not excessive, considering the number of bacteria in saliva of periodontitis patients (26–28). Furthermore, we were unable to identify the pathogenic gut bacteria involved in aggravation of NAFLD. Therefore, further studies are needed to identify the bacteria responsible for the exacerbation of NAFLD caused by *P. gingivalis* administration.

## Data Availability Statement

The datasets presented in this study can be found in online repositories. The names of the repository/repositories and accession number(s) can be found below: https://www.ncbi.nlm.nih.gov/, GSE136937.

## Ethics Statement

The animal study was reviewed and approved by The Institutional Animal Care and Use Committee of Niigata University (permit number; SA00328).

## Author Contributions

KyY generated data and wrote the manuscript. TK, YT, KS, MN, MY-T, MY-H, TT, and AM generated data. TK, NM, EM, WS, JK, and SO analyzed the data. NT, KT, JK, and HO contributed to the discussion. KaY planned the study and wrote the manuscript. All authors had access to the study data and reviewed and approved the final manuscript.

## Funding

This work was supported by JSPS KAKENHI [JP15H02578 and JP18H04067 (to KaY), and JP16H05207 to HO)], the Japan Agency for Medical Research and Development-Core Research for Evolutional Science and Technology (JP18gm0710009 to HO) and Sunstar Inc. (to KaY).

## Conflict of Interest

The authors declare that the research was conducted in the absence of any commercial or financial relationships that could be construed as a potential conflict of interest.

## Publisher’s Note

All claims expressed in this article are solely those of the authors and do not necessarily represent those of their affiliated organizations, or those of the publisher, the editors and the reviewers. Any product that may be evaluated in this article, or claim that may be made by its manufacturer, is not guaranteed or endorsed by the publisher.
