# Supplementary material for: Oral Pathobiont-Induced Changes in Gut Microbiota Aggravate the Pathology of Nonalcoholic Fatty Liver Disease in Mice
Source: Front Immunol. 2021 Oct 11;12:766170. doi: 10.3389/fimmu.2021.766170 (PMC8543001; doi:10.3389/fimmu.2021.766170)
Supplement: Supplementary file 1 [file DataSheet_1.pdf]

Supplementary Figure 1

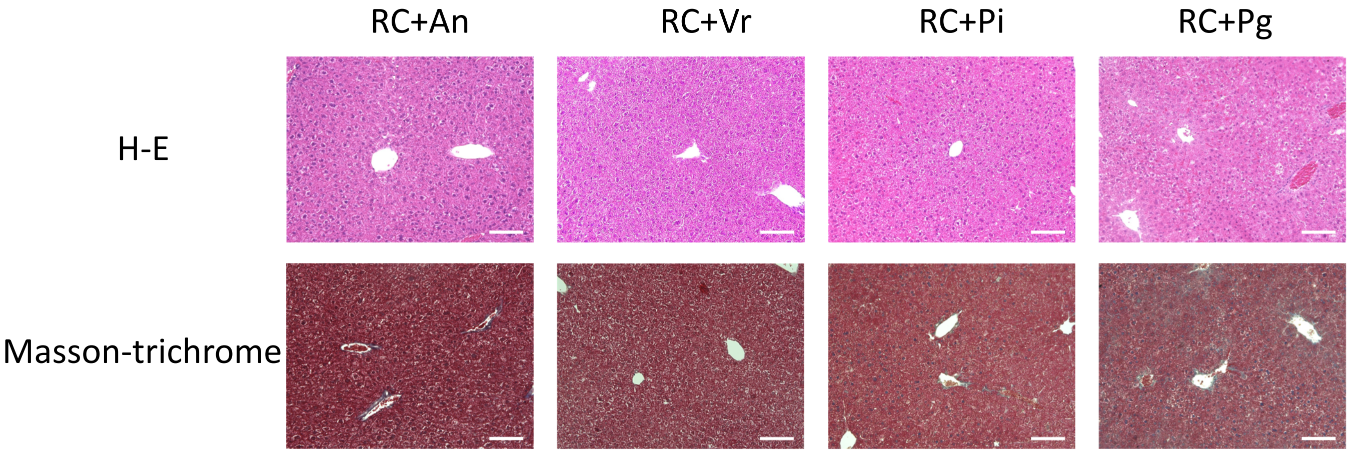

Supplementary Fig. 1. Administration of commensals and Pi do not induce liver pathology in RC-fed mice. Hematoxylin and eosin (upper panels), and Masson's trichrome staining (lower panels) of liver scale bars, 100 µm)

Supplementary Figure 2

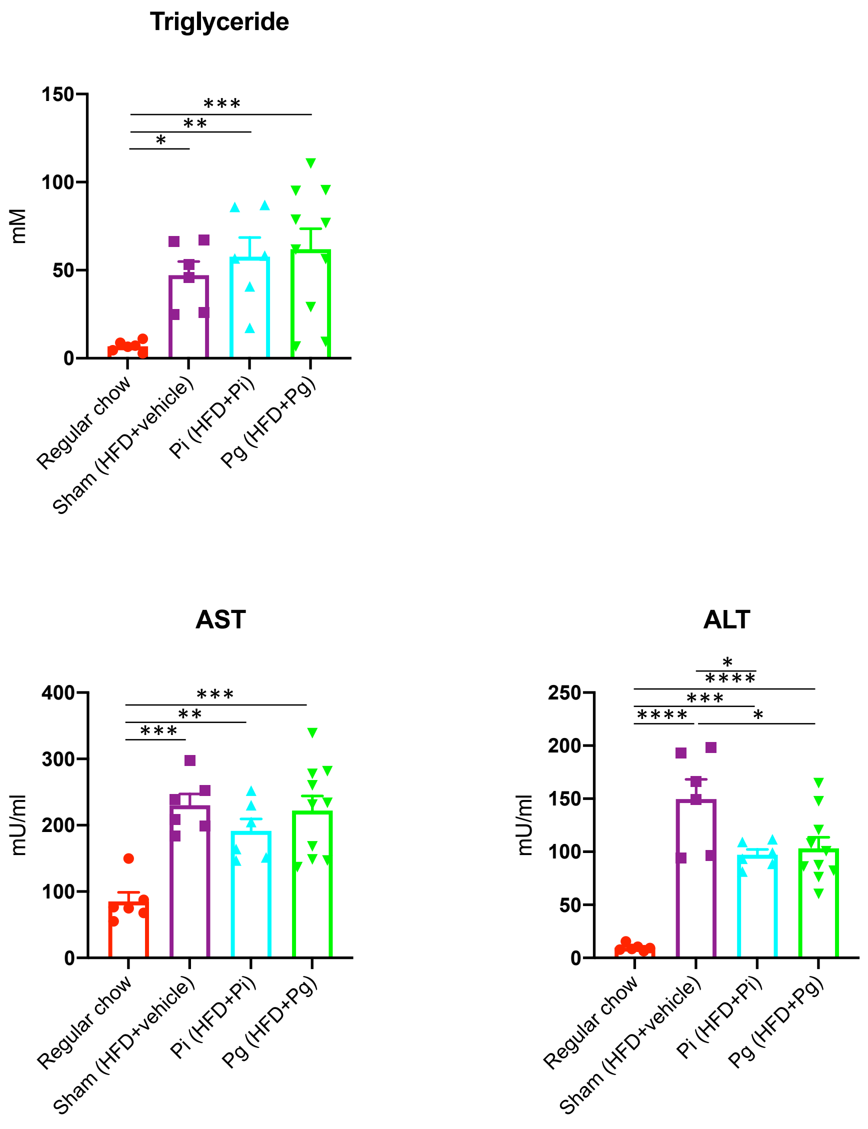

Supplementary Fig. 2. Effect of bacterial administration on hepatic content of triglyceride, and serum levels of AST and ALT in high fat diet-fed mice (n=6-10/group) . RC: C57BL/6J mice fed regular chow; Sham: Mice fed CDAHFD60 plus sham administration; Pi: Mice fed CDAHFD60 plus *P. intermedia* administration; Pg: Mice fed CDAHFD60 plus *P. gingivalis* administration. Data are expressed as the mean ± s.e.m. (C). *$P < 0.05$, ** $P < 0.005$, *** $P < 0.001$, and **** $P < 0.0001$; one-way ANOVA with Tukey's correction for multiple comparisons.

Supplementary Figure 3

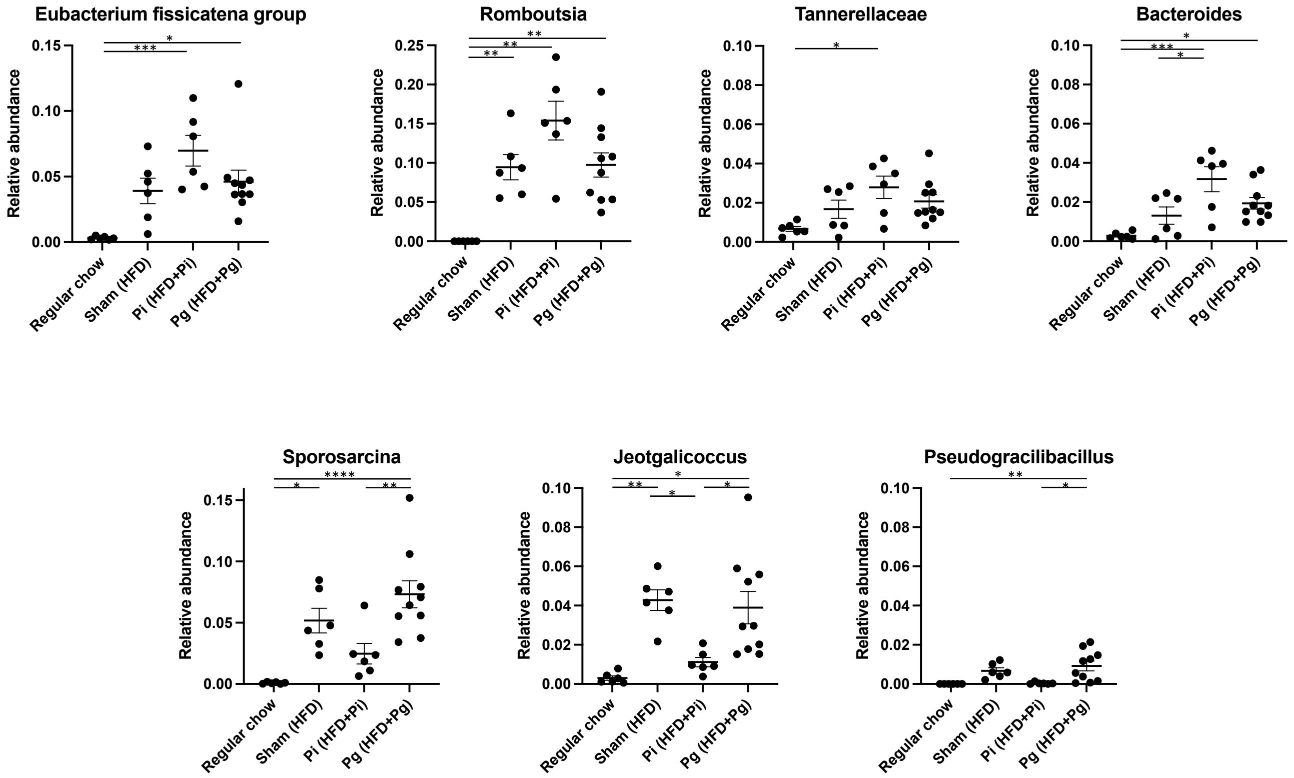

Supplementary Fig. 3. Effect of CDAHFD60 feeding and subsequent bacterial administration on the gut microbiota composition (n=6-10/group). Fecal samples from mice that received the various treatment were subjected to 16S rRNA sequencing. Relative abundances of characteristic genera not shown in Figure 5D in each experimental group at TP3 . Data are expressed as the mean ± s.e.m. P values were calculated using one-way ANOVA with Tukey's multiple comparisons test. *P < 0.05, **P < 0.005, ***P < 0.001, ****P < 0.0001.

Supplementary Figure 4

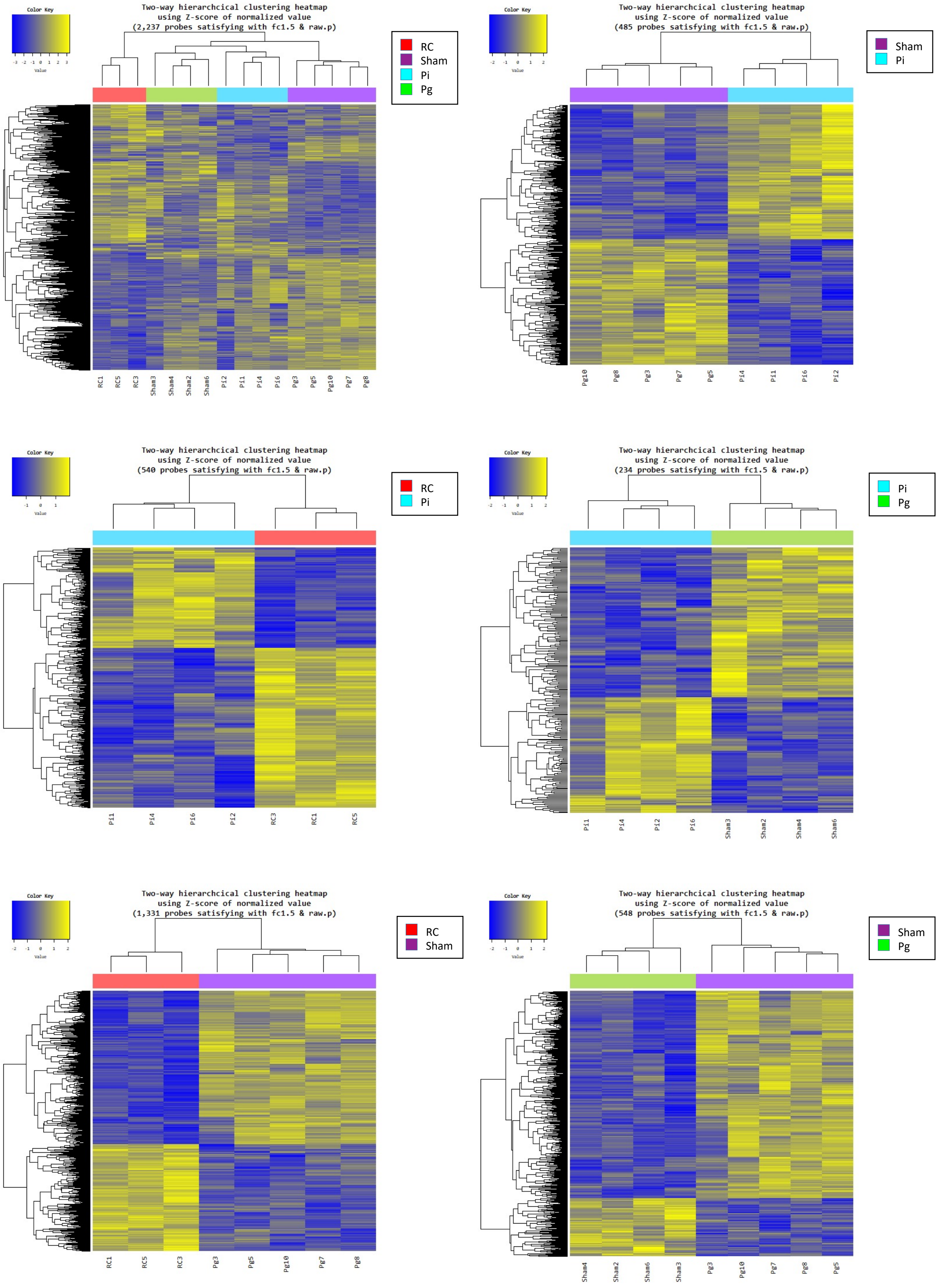

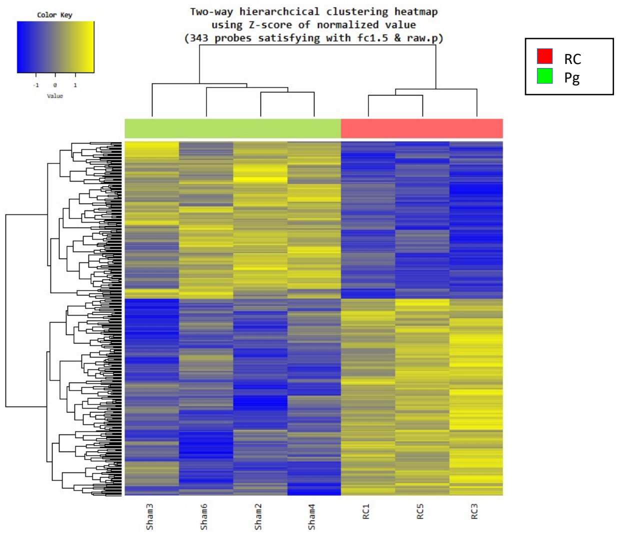

Supplementary Fig. 4. Hierarchical clustering heatmap of differentially expressed genes in the gut. Differentially expressed genes (DEGs) are listed along the Y-axis in the order that they clustered as indicated by the colored line along the Y-axis (n=3-5/group). Each column contains expression values for an individual animal with groups indicated along the X-axis, and clustering indicated by the dendrogram above the figure. Compared groups are indicated at the top left in each panel.

Supplementary Figure 5

(A)

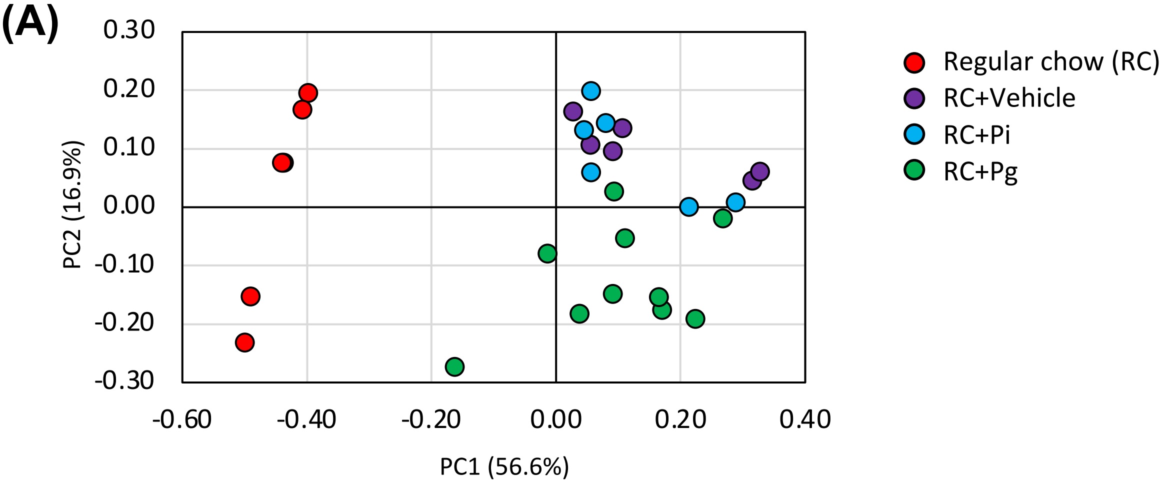

(B)

	1.RC	2.Sh	3.Pi	4.Pg
1.RC	19	0	0	0
2.Sh	0	2	8	5
3.Pi	0	15	4	0
4.Pg	0	1	6	25

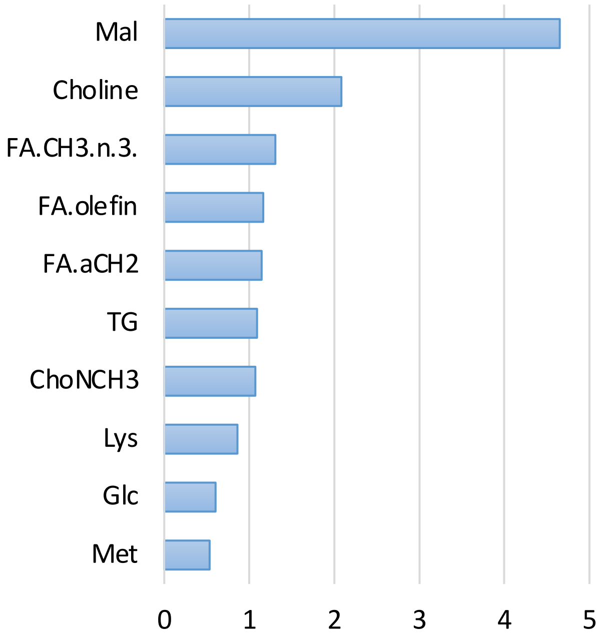

(C)

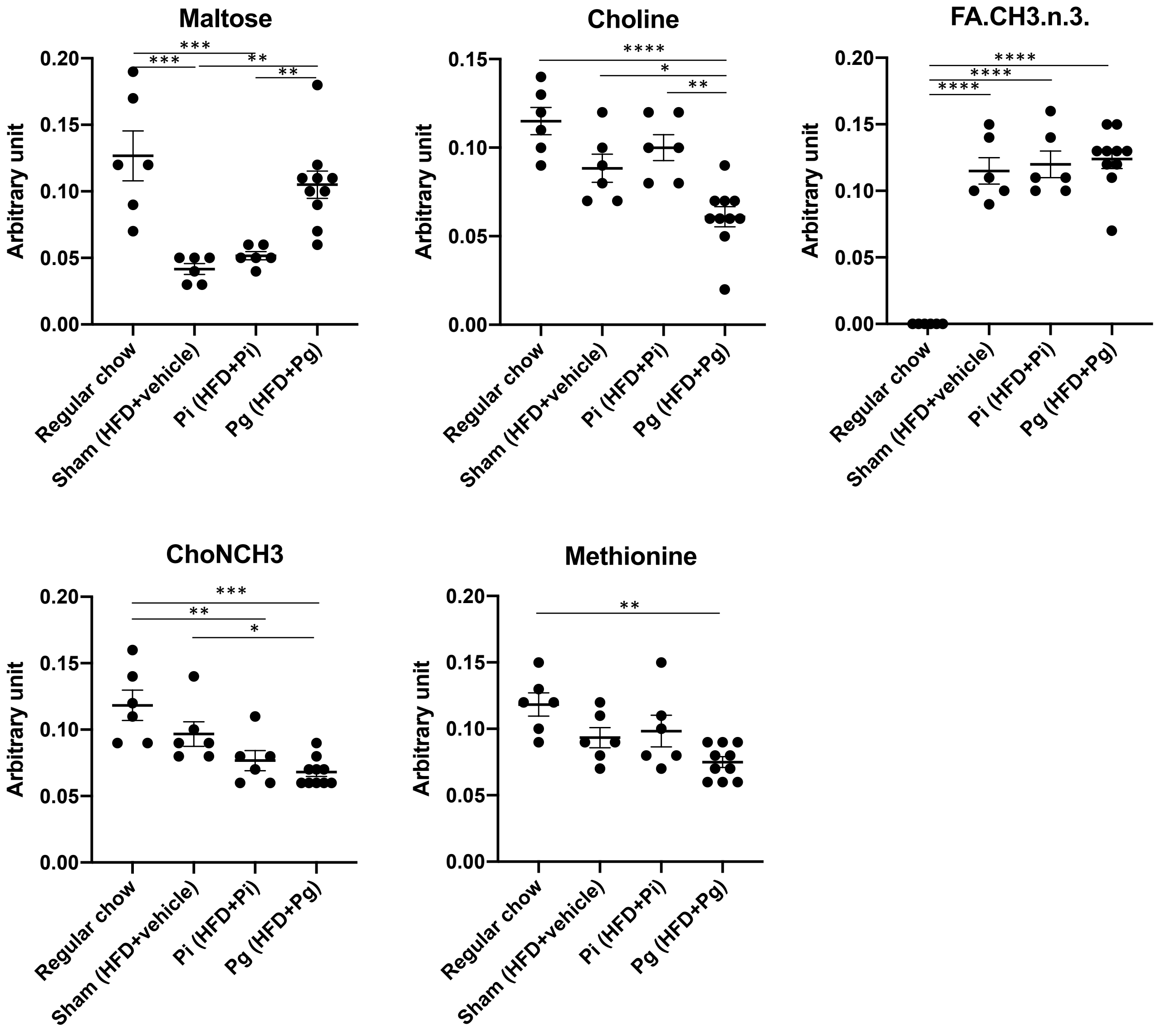

Supplementary Figure 5. Effect of CDAHFD60 feeding and subsequent bacterial administration on metabolites in liver tissue.

(A) PCA of tissue metabolites in each group. (B) Machine learning (random forest) classification of each group. Left: confusion matrix (RC: regular chow; Sham: HFD + vehicle; Pi: HFD + Pi; Pg: HFD + Pg). Right: important variables (metabolites) contributing to four classifications. Abbreviations of metabolites are as follows: Mal: maltose; FA: fatty acid, TG: triglyceride; CHO: choline; Lys: Lysine, Glc: glucose; Met: methionine. (C) Compounds that differed in abundance among groups (n=6-10/group). Data are expressed as the mean ± s.e.m. *P* values were calculated using one-way ANOVA with Tukey's multiple comparisons test. $^{**}P < 0.005$, $^{***}P < 0.001$, and $^{****}P < 0.0001$.

Supplementary Figure 6

A

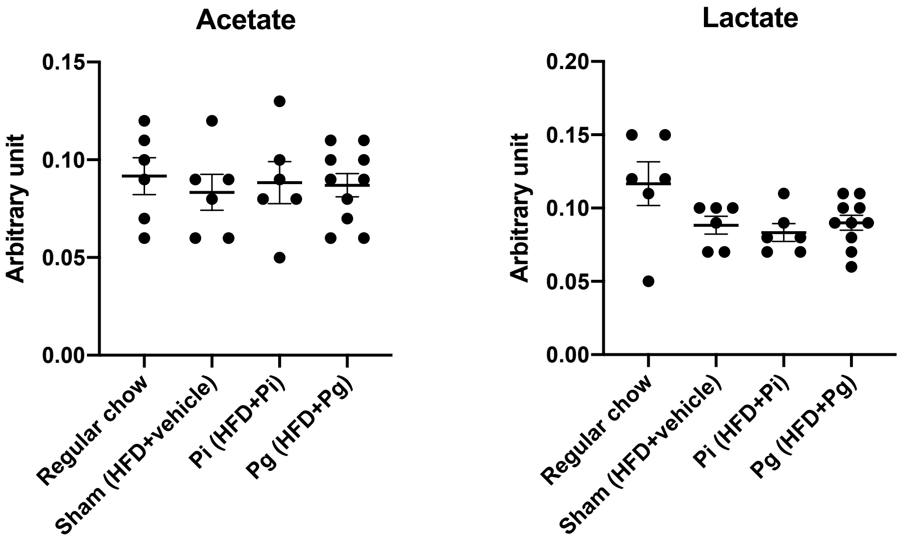

B

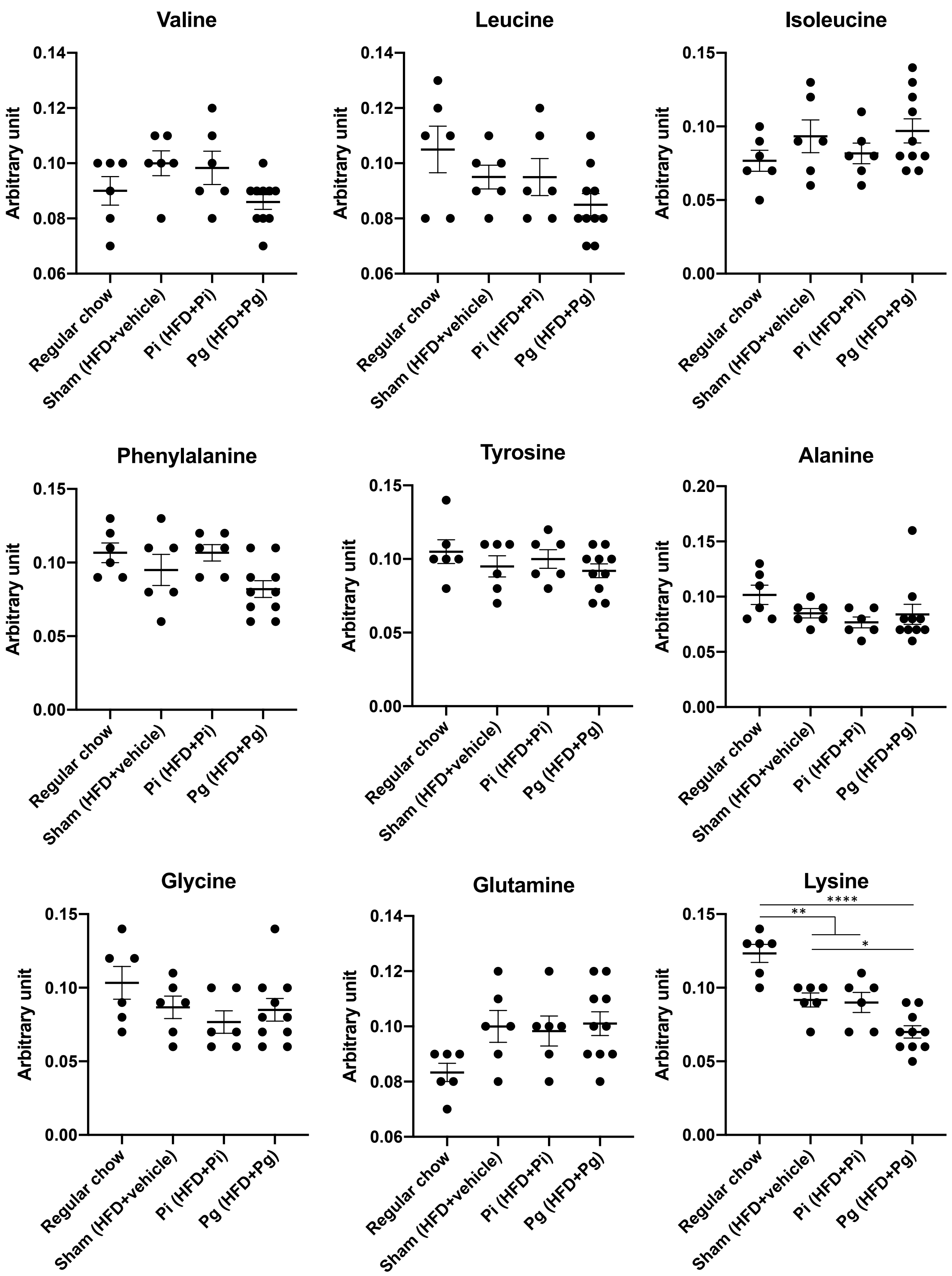
Valine
Leucine
Isoleucine
Phenylalanine
Tyrosine
Alanine
Glycine
Glutamine
Lysine
Arbitrary unit
Regular chow
Sham (HFD+vehicle)
Pi (HFD+Pi)
Pg (HFD+Pg)
****
**
*

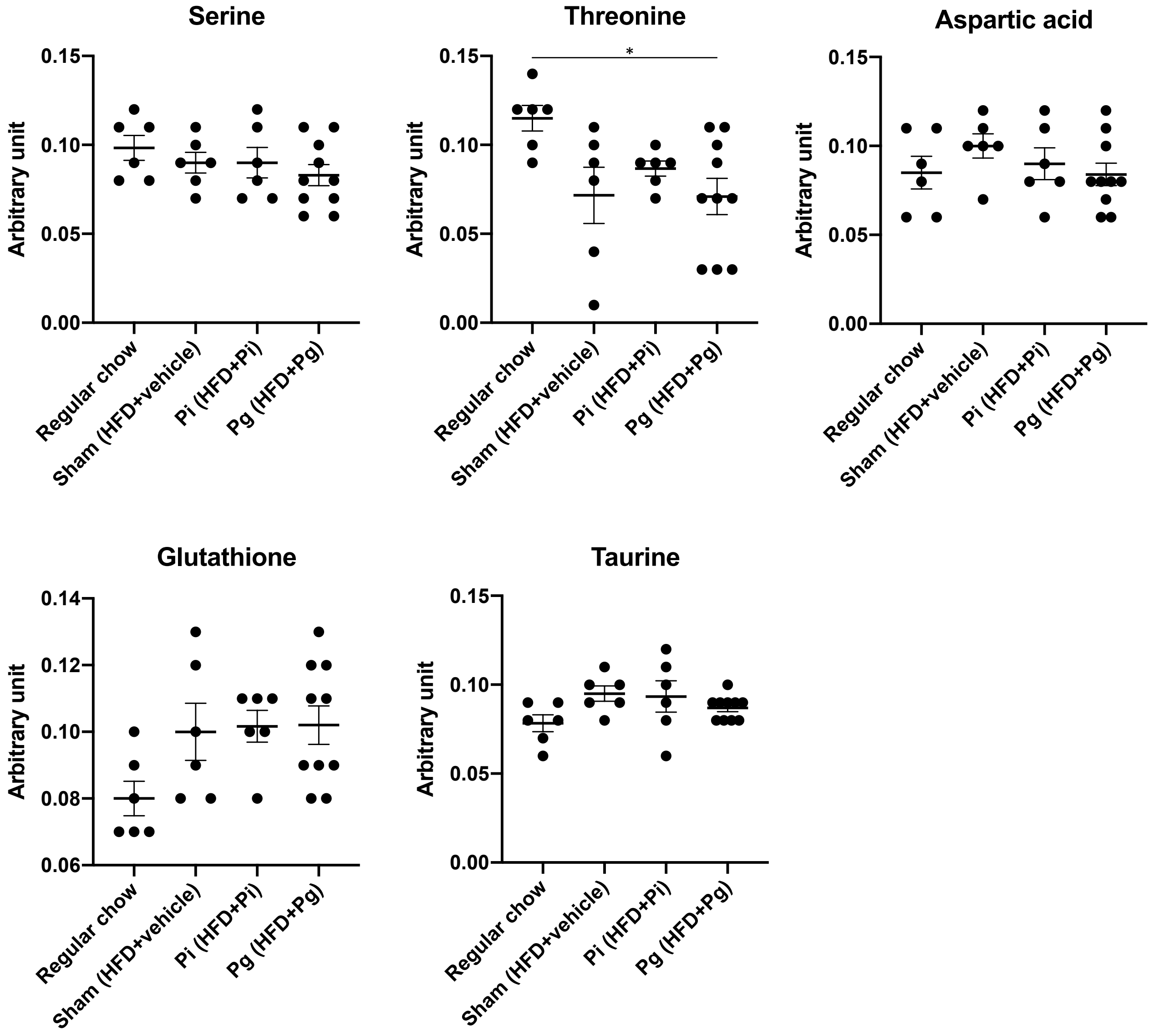
Serine
Threonine
Aspartic acid
Glutathione
Taurine
Arbitrary unit
0.15
0.10
0.05
0.00
0.14
0.12
0.10
0.08
0.06
*
Regular chow
Sham (HFD+vehicle)
Pi (HFD+Pi)
Pg (HFD+Pg)

C

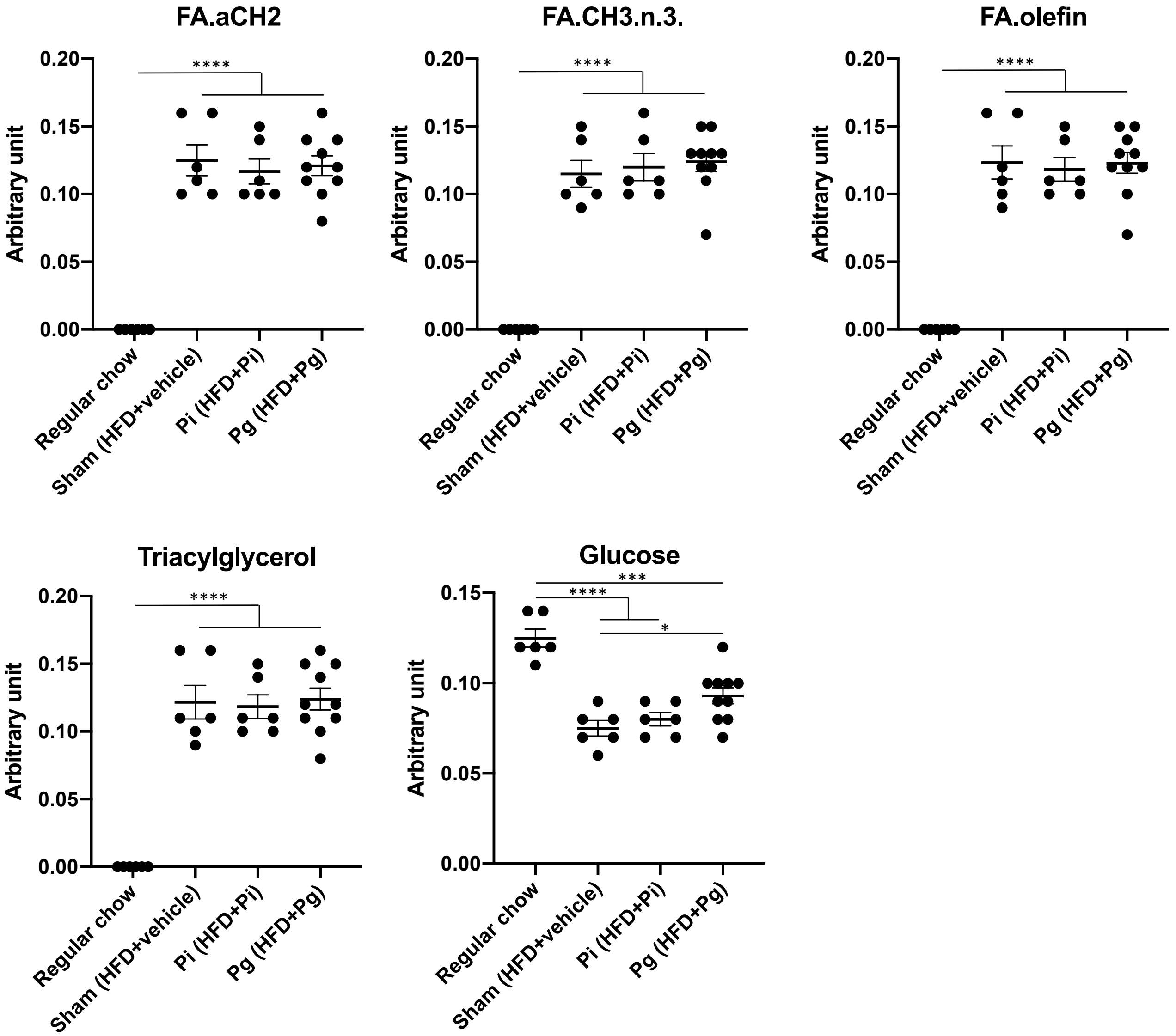

Supplementary Fig. 6. Effect of CDAHFD60 feeding and subsequent bacterial administration on metabolites in the liver tissue (n=6-10/group).
(A) Short chain fatty acids and (B) amino acids that differed in abundance among groups.
(C) Other metabolites that differed in abundance among the groups. Data are expressed as the mean ± s.e.m. **P* < 0.05, ***P* < 0.005, ****P* < 0.001, and *****P* < 0.0001; one-way ANOVA with Tukey's correction for multiple comparisons.

Supplementary Figure 7

A

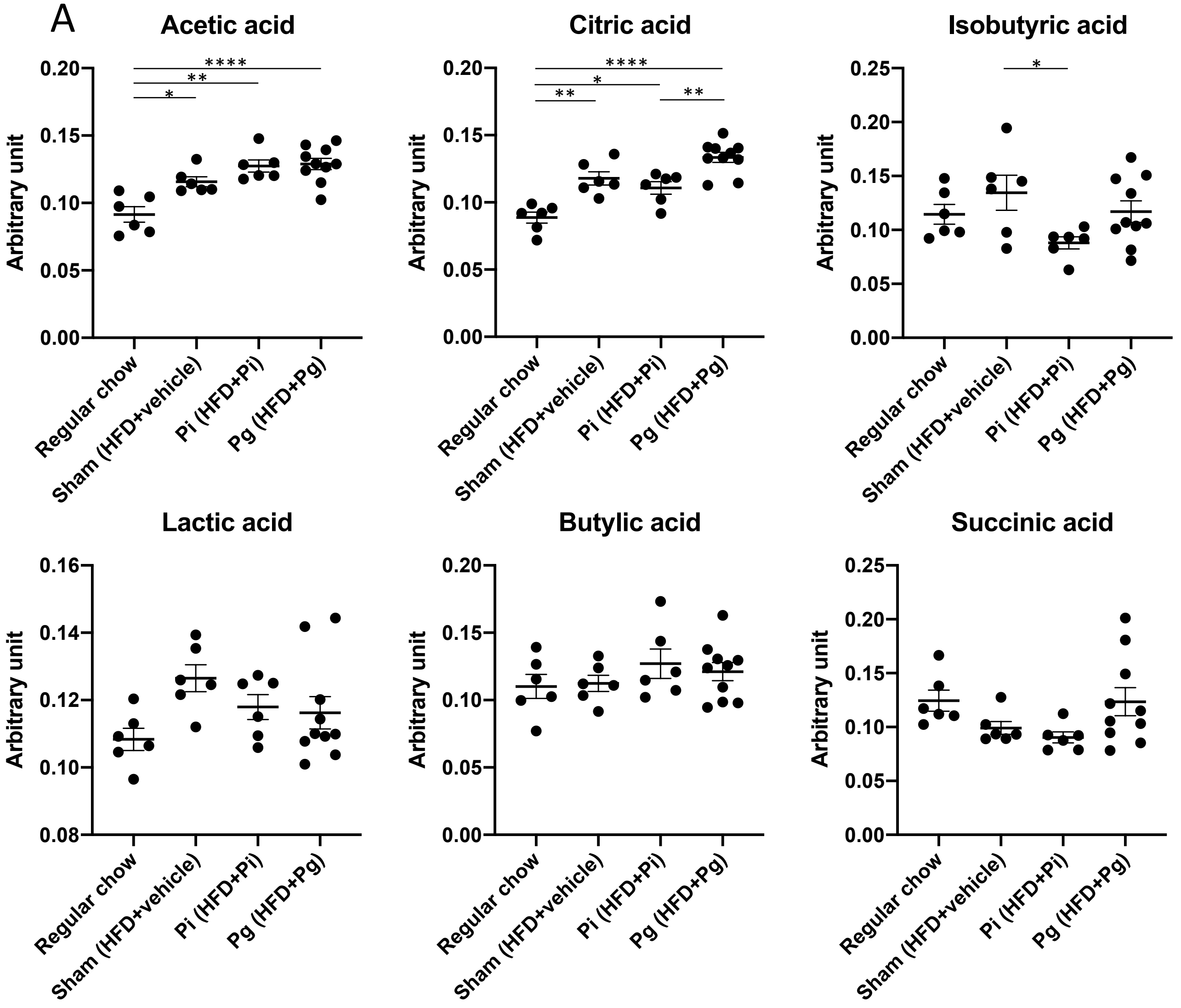

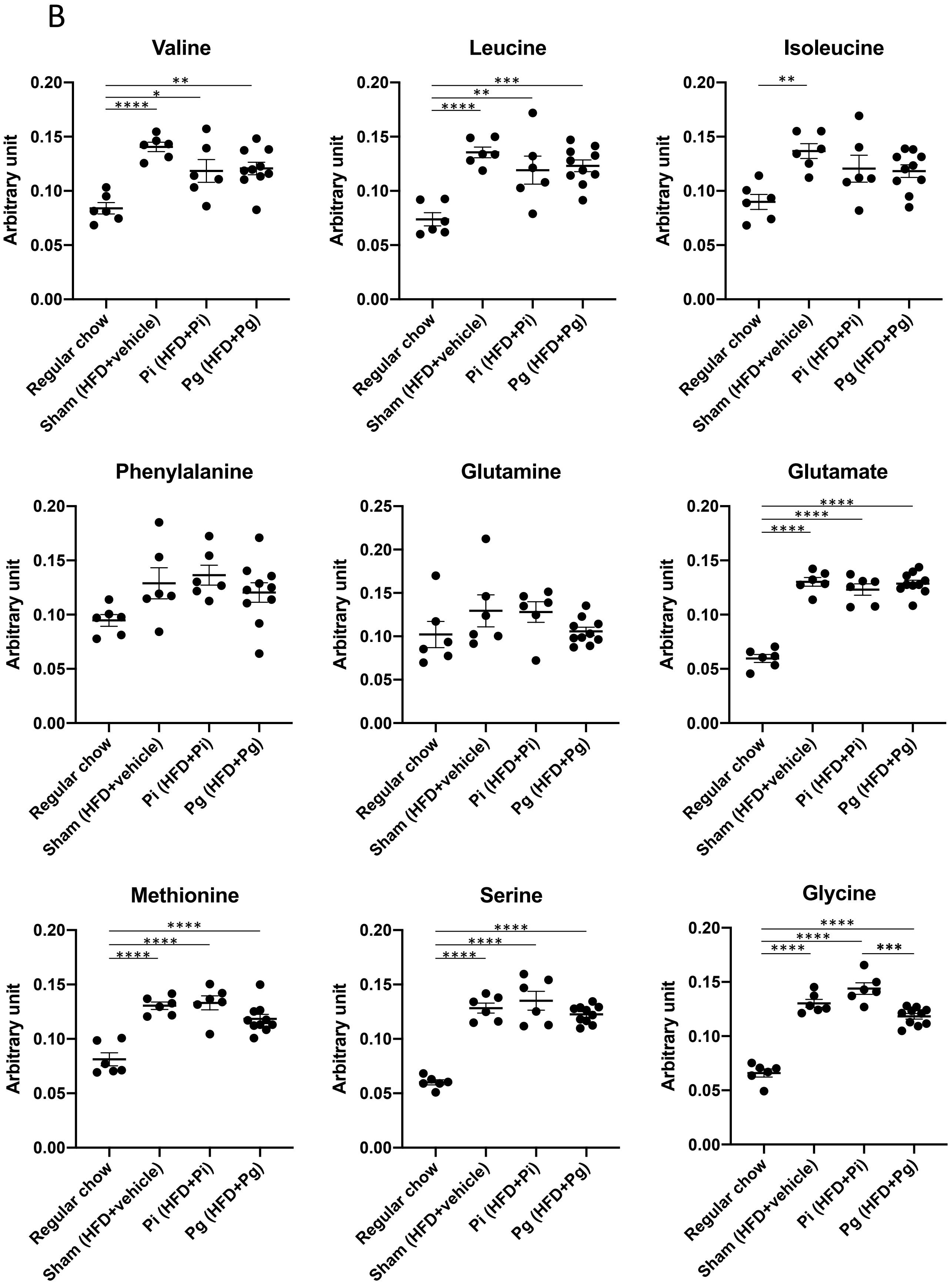
B
Valine
Leucine
Isoleucine
Phenylalanine
Glutamine
Glutamate
Methionine
Serine
Glycine
Arbitrary unit
Regular chow
Sham (HFD+vehicle)
Pi (HFD+Pi)
Pg (HFD+Pg)

C

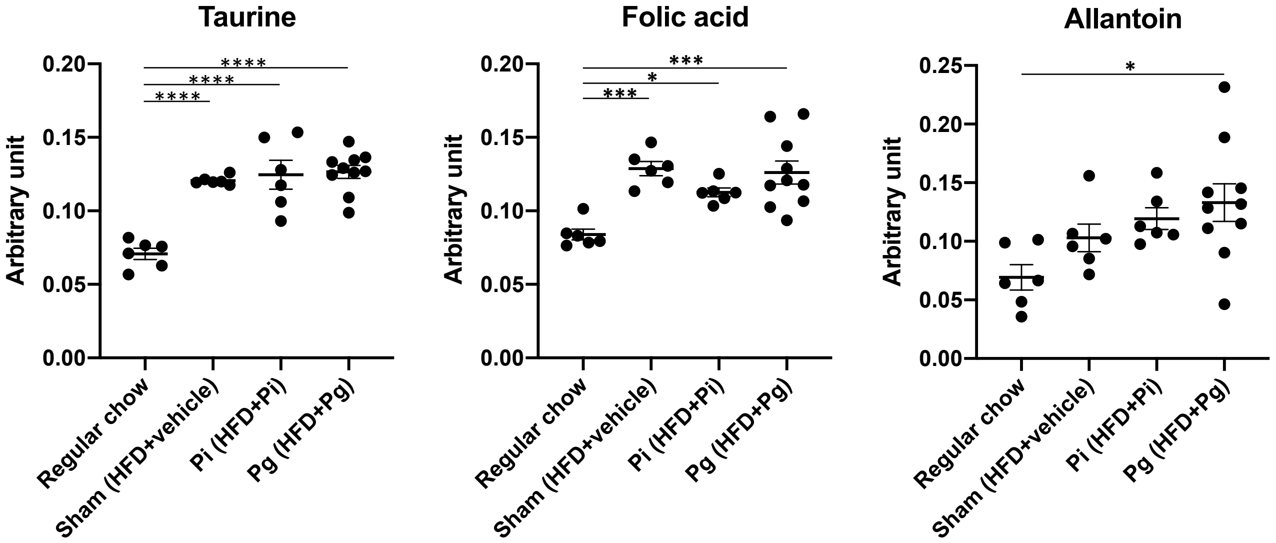

Supplementary Fig. 7. Effect of CDAHFD60 feeding and subsequent bacterial administration on serum metabolites (n=6-10/group).
(A) Serum short chain fatty acids and (B) amino acids that differed in abundance among groups. (C) Other metabolites that differed in abundance among the groups. Data are expressed as the mean ± s.e.m. *$P < 0.05$, **$P < 0.005$, ***$P < 0.001$, and ****$P < 0.0001$; one-way ANOVA with Tukey's correction for multiple comparisons.

Supplementary Figure 8

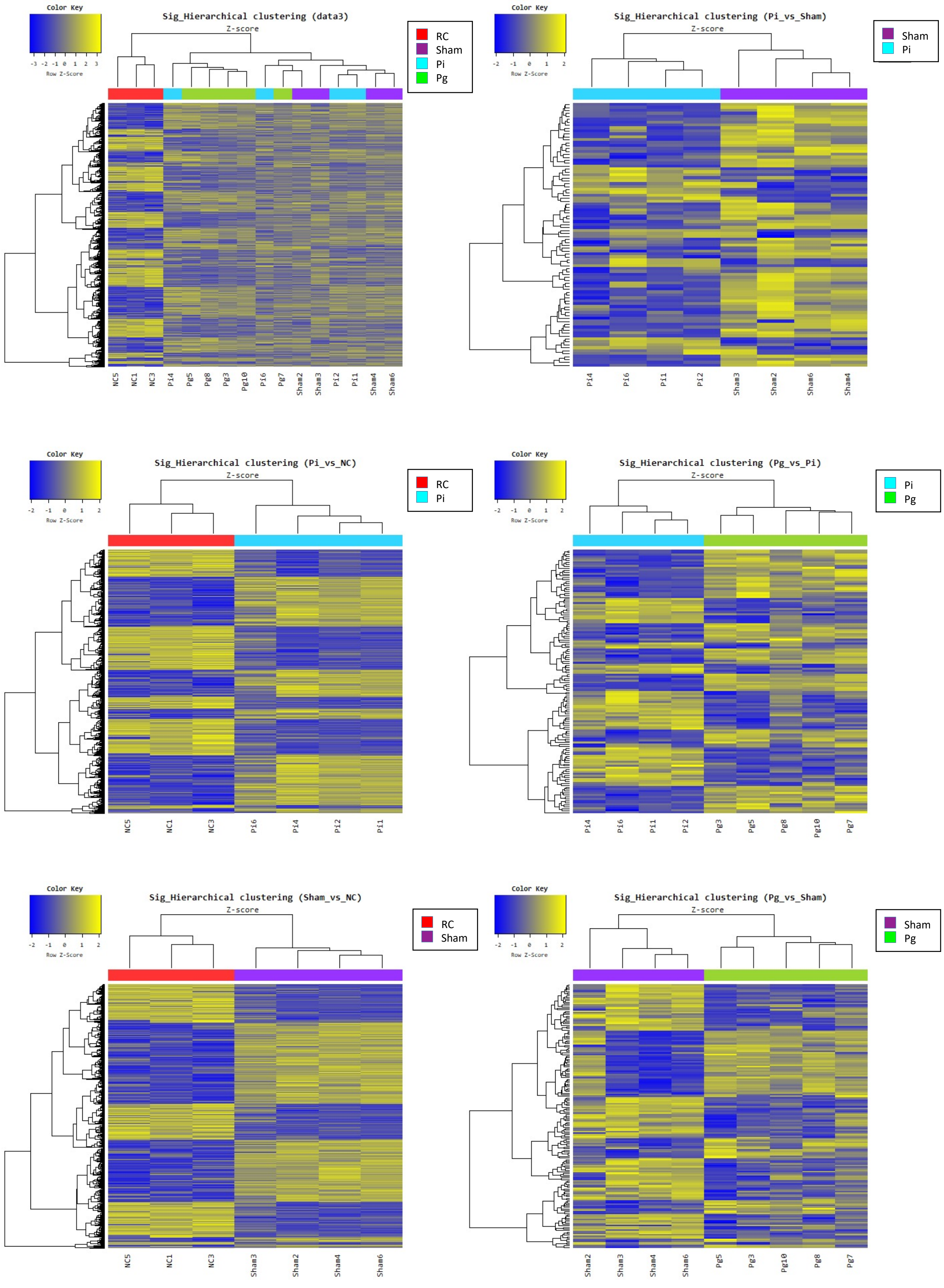

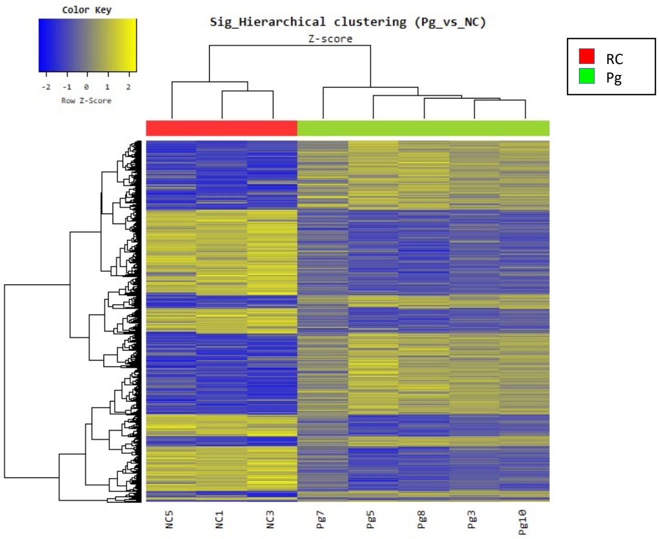

Supplementary Fig. 8. Hierarchical clustering heatmap of differentially expressed genes in the liver. Differentially expressed genes (DEGs) are listed along the Y-axis in the order that they clustered as indicated by the colored line along the Y-axis (n=3-5/group). Each column contains expression values for an individual animal with groups indicated along the X-axis, and clustering indicated by the dendrogram above the figure. Compared groups are indicated at the top left in each panel.

Supplementary Fig. 6 continued

Supplementary Figure 9

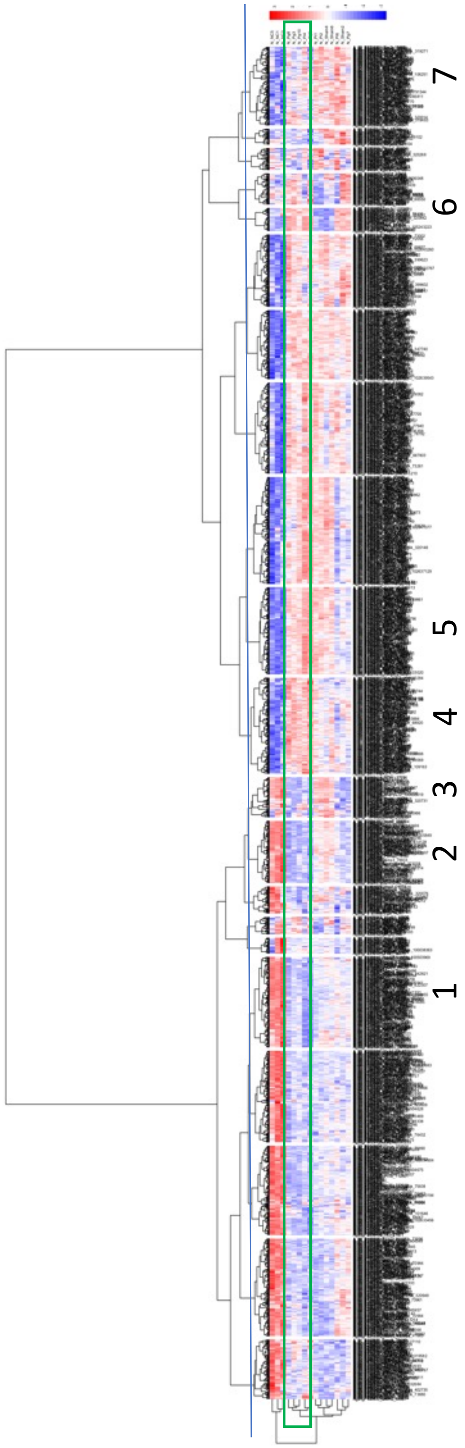

Supplementary Fig. 9. Dendrogram and heatmap for gene expression in the liver at TP3.
A blue line was drawn and the dendrogram was divided into 21 clusters in order of the groups of genes showing a similar expression pattern belonging to the same cluster. The green square shows the P. gingivalis-administered group. We selected seven clusters as indicated by the number assignment in the figure with a characteristic expression pattern.

Supplementary Figure 10

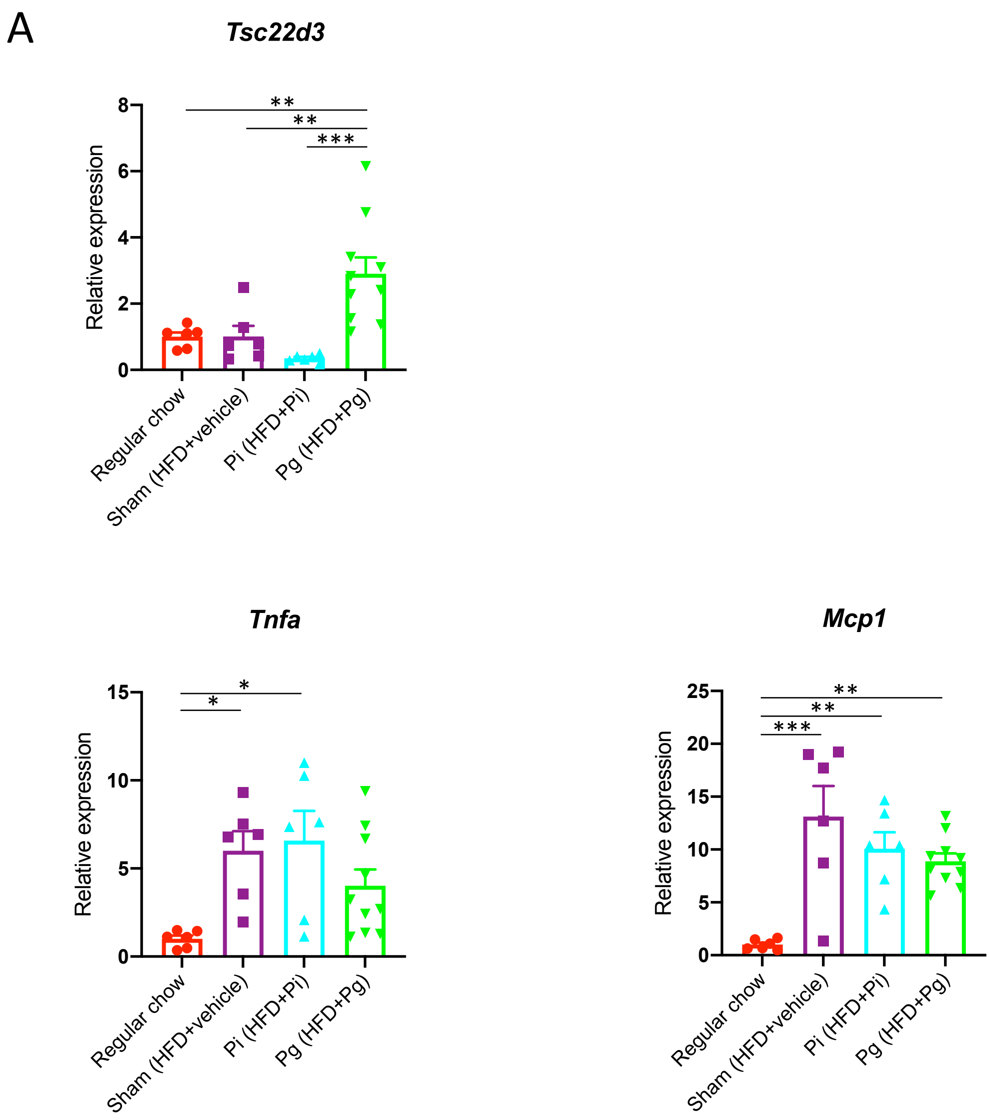

Supplementary Fig. 10

B

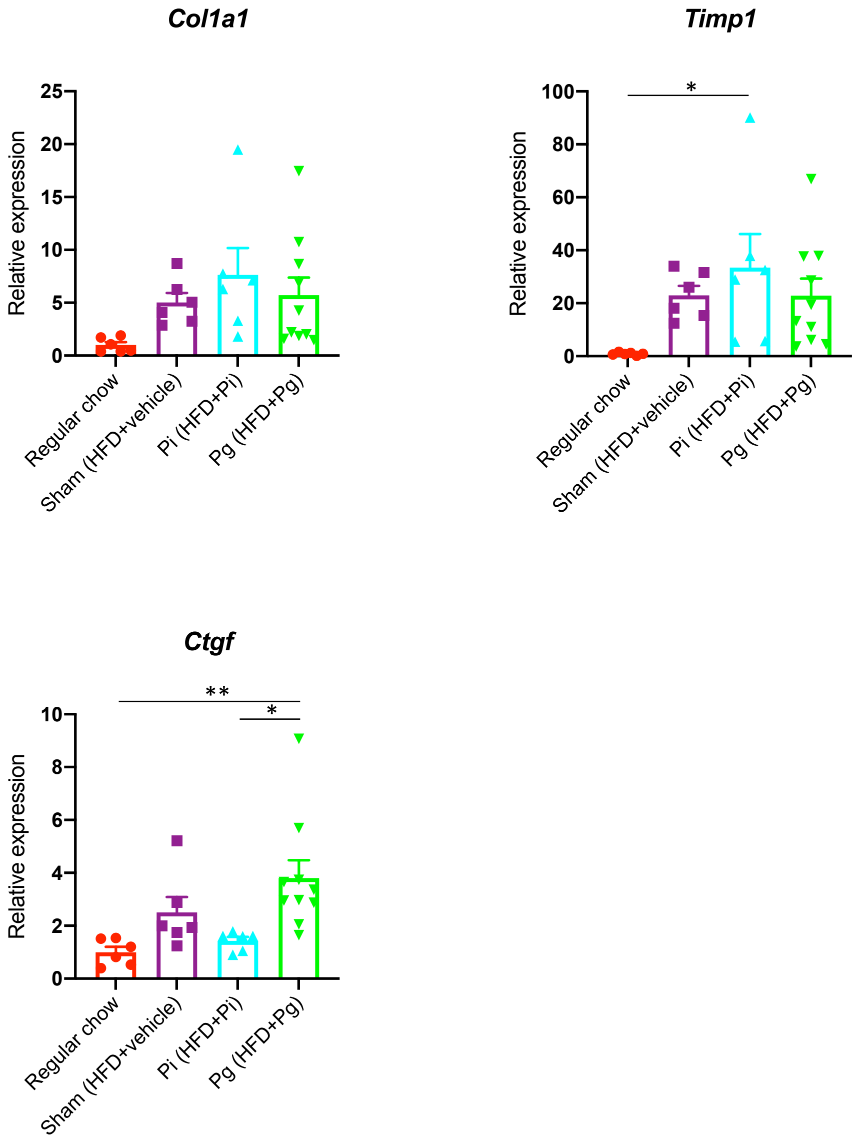

Supplementary Fig. 10

C

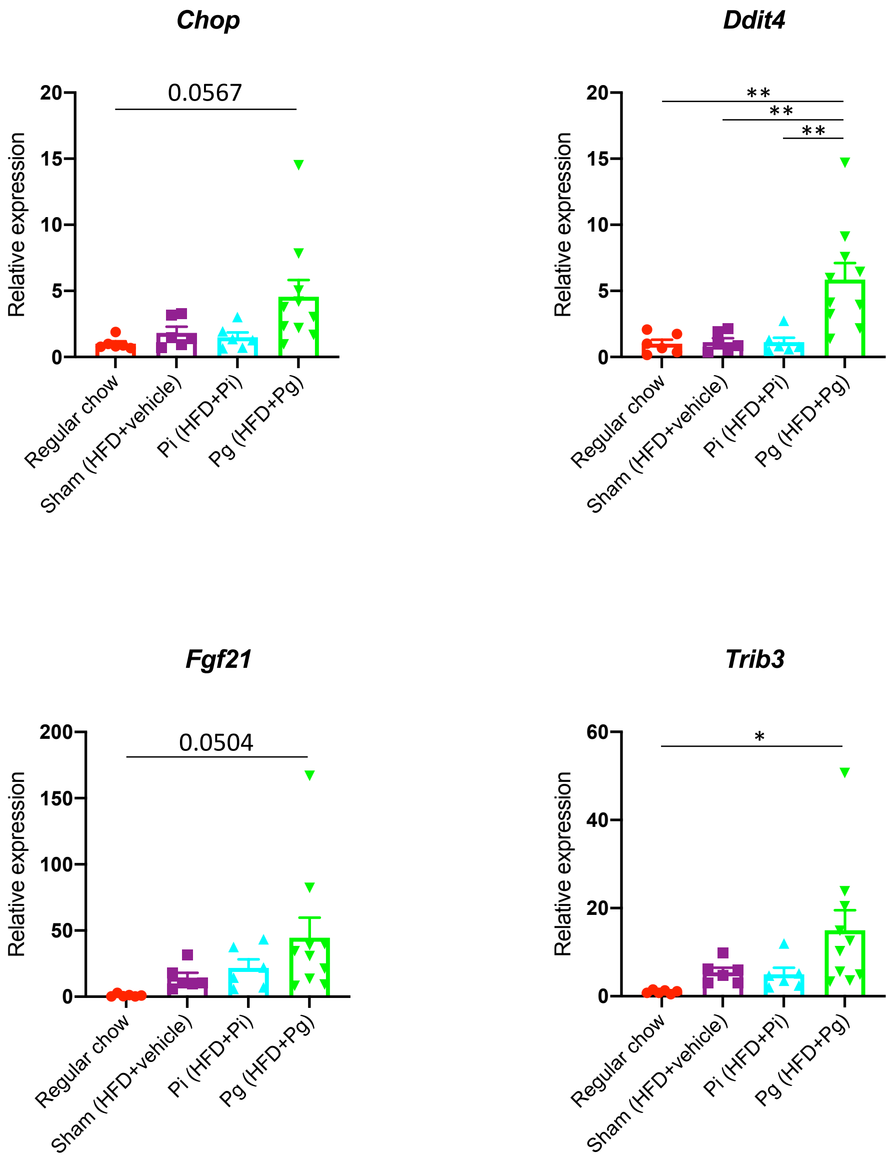

Supplementary Fig. 10

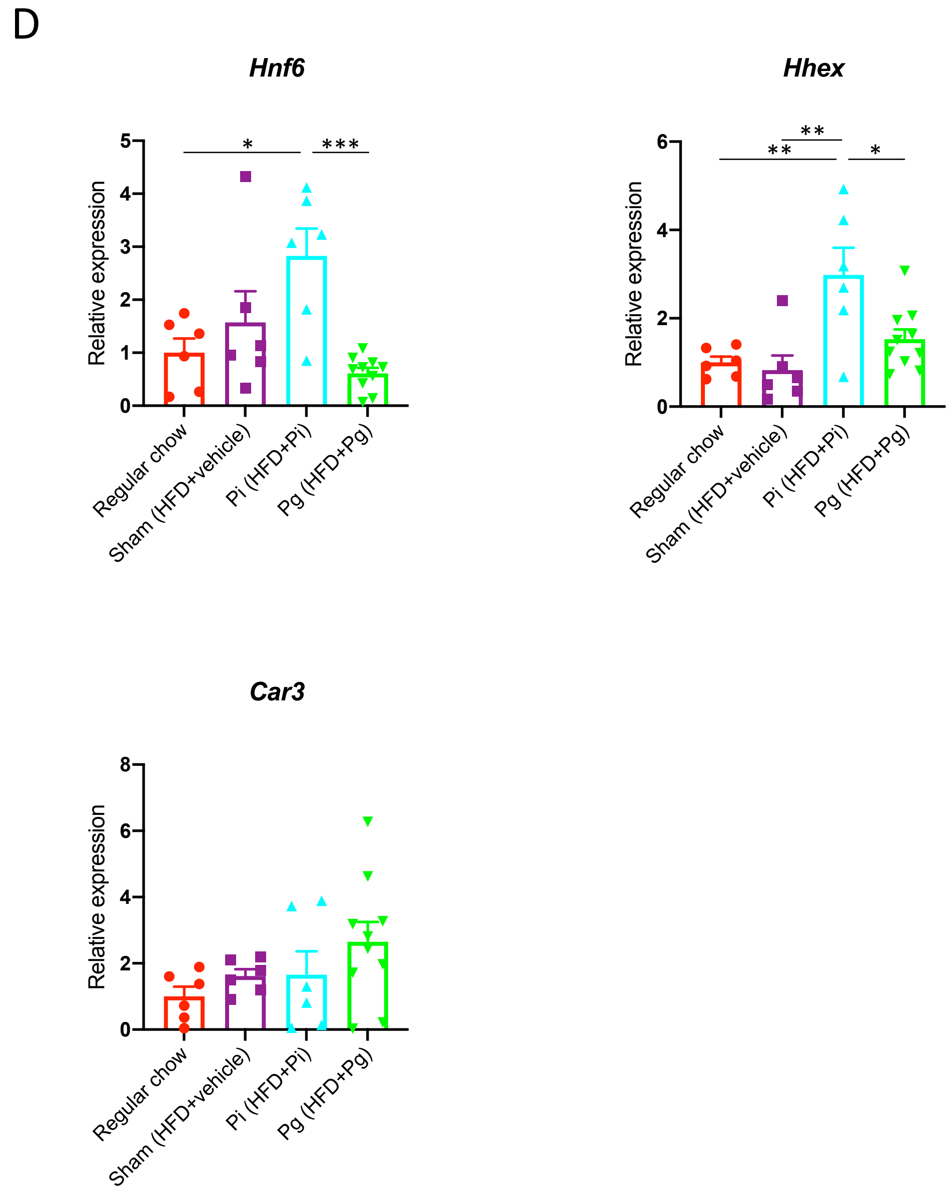

Supplementary Fig. 10

E

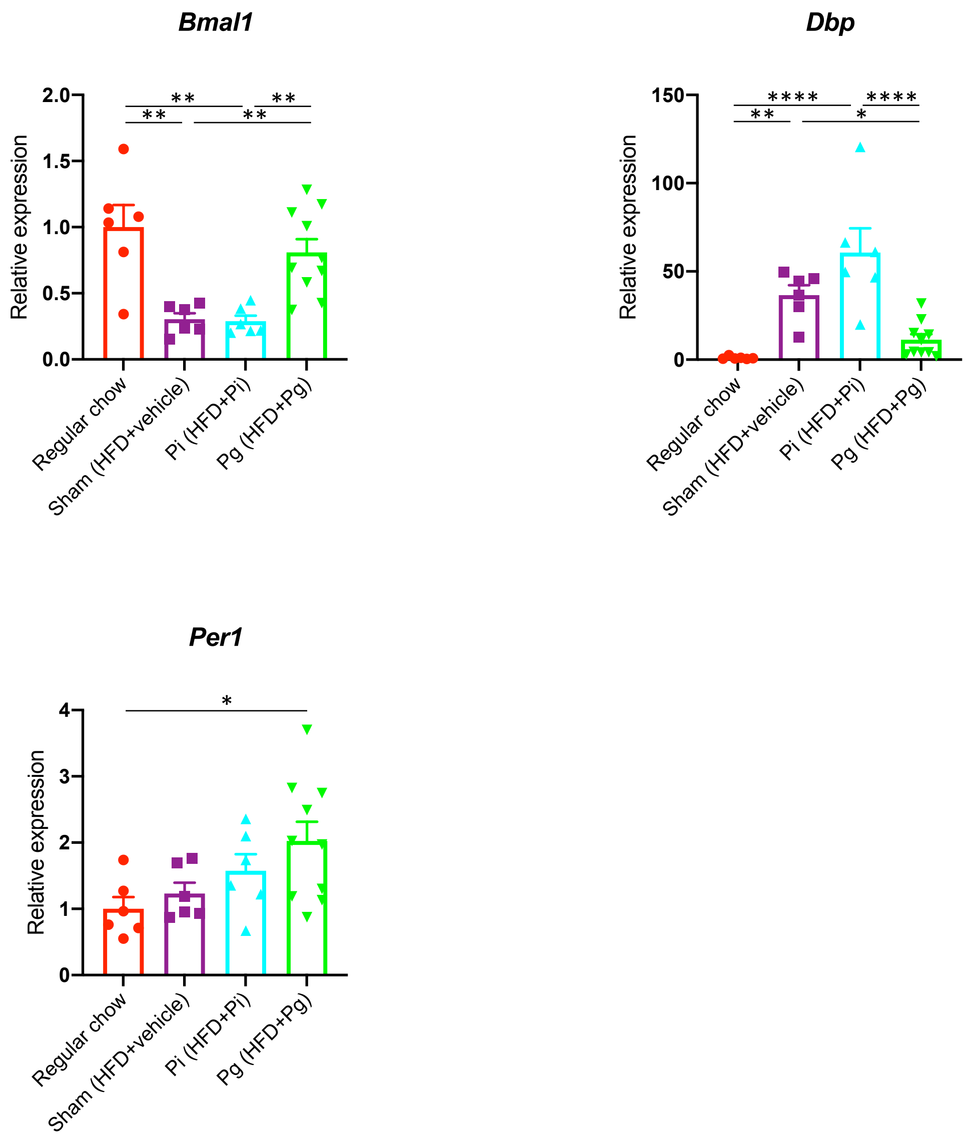

Supplementary Fig. 10. CDAHFD60 feeding and subsequent bacterial administration affects the expression of inflammatory genes. (A) inflammatory genes, (B) fibrosis-related genes, (C) ER-stress-related genes, (D) carcinogenesis-related genes, and (E) circadian rhythm-related genes (n=6-10/group). Data are expressed as the mean ± s.e.m. * $P < 0.05$, ** $P < 0.005$, and *** $P < 0.001$; one-way ANOVA with Tukey's correction for multiple comparisons .

Supplementary Figure 11

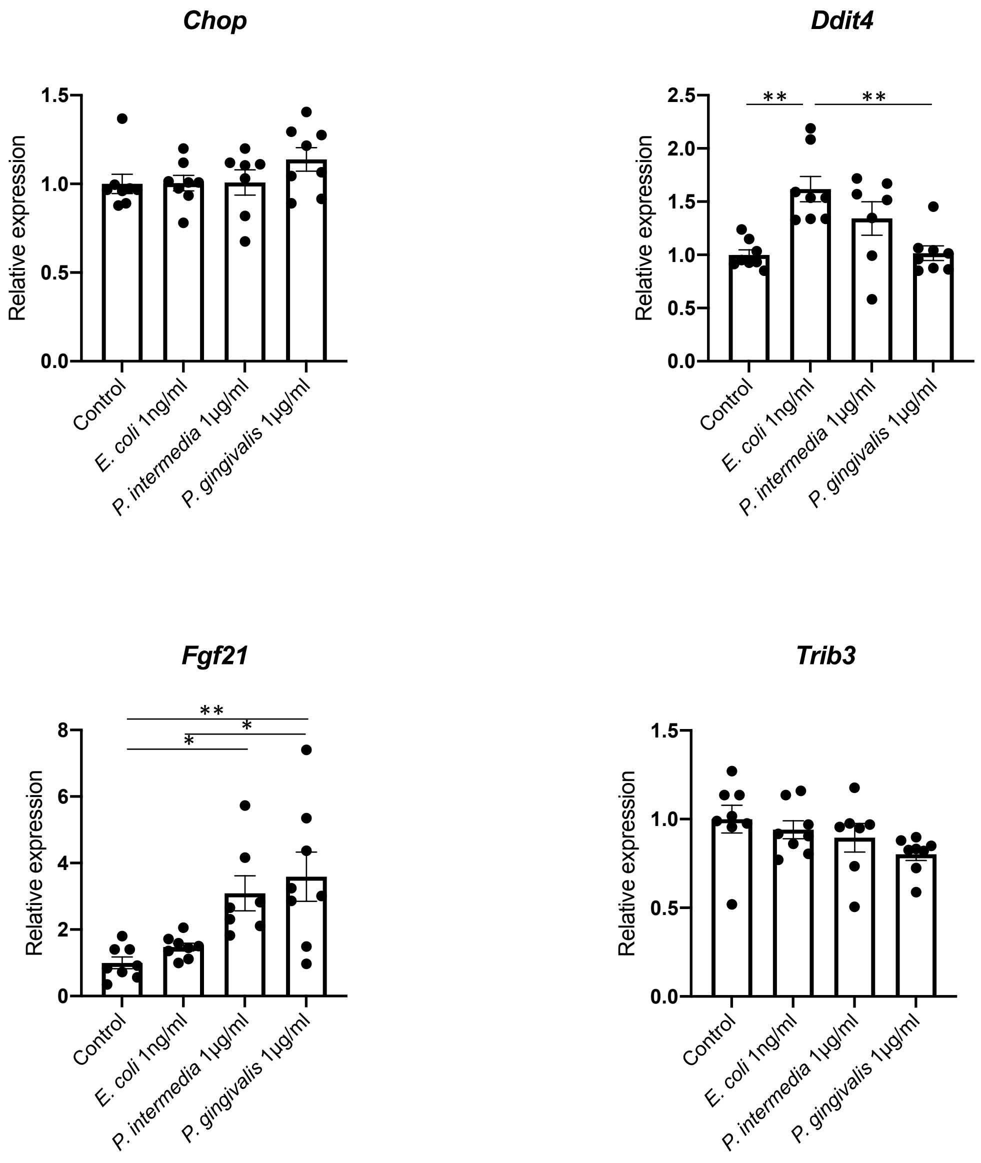

Supplementary Fig. 11. Effect of LPS stimulation on ER stress-associated genes in HepG2 cells (n=8/group). HepG2 cells treated with FFA were stimulated with LPS derived from *E. coli*, *P. intermedia*, or *P. gingivalis* at the indicated doses for 4 h. Relative expression of the indicated genes is shown. Data are expressed as the mean ± s.e.m. *: $P < 0.05$, ** $P < 0.01$; one-way ANOVA with Tukey's correction for multiple comparisons.
