# Supplementary material for: Oral Pathobiont-Induced Changes in Gut Microbiota Aggravate the Pathology of Nonalcoholic Fatty Liver Disease in Mice
Source: Front Immunol. 2021 Oct 11;12:766170. doi: 10.3389/fimmu.2021.766170 (PMC8543001; doi:10.3389/fimmu.2021.766170)
Supplement: Supplementary file 2 [file Table_1.docx]

**Supplementary Tables**

Supplementary Table 1 Gene lists used for DAVID Enrichment analysis

| Cluster 1 (124 gene) |
| --- |
| 1700029P11Rik, 1810008I18Rik, 4732465J04Rik, 4930504D19Rik, 5830473C10Rik, 9330151L19Rik, 9330175M20Rik, AI182371, Abcd3, Abhd6, Acadsb, Acsl1, Adck5, Adgrf1, Adipor2, Agmo, Atp5f1, Baat, Baiap2l2, C6, C8b, Camp, Car1, Ccin, Cd4, Cfap54, Chchd7, Coq7, Cox6b1, Cyp27a1, Cyp2c29, Cyp2c38, Cyp2c44, Cyp2c70, Cyp2d34, Cyp2d9, Cyp4a12a, Cyp4a12b, Cyp7b1, Dct, Duox1, E330032C10Rik, Ebp, Echdc1, Egfr, Fhit, Gabrr1, Ghr, Gm14151, Gm15517, Gm3285, Gm32894, Gm33874, Gnas, Hsbp1l1, Hsd3b1, Hsd3b2, Hsd3b3, Hsd3b5, Hsd3b6, Igdcc3, Igf1, Kazn, Keg1, Krtap5-5, Kynu, Lama3, Lifr, Lin28a, Lmx1b, Lrrc10b, Lrrc51, Mab21l2, Marc1, Mdh1, Mettl20, Mettl7a1, Mttp, Mup11, Mup21, ND5, Ngf, Nqo2, Olfr125, Olfr1535, Parvb, Pax2, Pnpla7, Prm3, Prn, Psd, Pyurf, Rab9, Retsat, Rmi2, Rybp, Sar1b, Serpina3c, Serpina3j, Serpina3k, Sh3tc2, Siglech, Slc22a28, Slc27a5, Slc35f4, Slc4a2, Slc4a9, Slco1a1, Sohlh2, Srd5a1, Sugct, Syt6, Tlcd2, Tmem91, Tmx4, Tnf, Tnfsf8, Tpm1, Ugt2a3, Ugt2b1, Ugt3a1, Zfp2, Zfp36l1, Zfp961 |
| Cluster 2 (70 genes) |
| 1700065D16Rik, 2210409E12Rik, 6430584L05Rik, A630001G21Rik, AB124611, Acaa1b, Amy2a5, Atp5h, Atp5o, Brwd3, Cacna2d1, Casp9, Cep170, Ces2b, Ces2c, Cnga1, Cox7c, Crx, Cryba4, Cyp2c53−ps, Disc1, Drp2, Ephx3, Fastkd1, Focad, G630064G18Rik, Gins3, Gm10765, Gm16386, Gm19412, Gm2138, Gm29782, Gm3704, Gm3772, Gm38596, Gm41795, Gm8479, Hsd17b2, Iqsec3, LOC101055849, Ly6g6c, Mdga1, Mdga2, Mup3, Nkain2, Nudt7, Nxnl1, Olfr1368, Olfr1466, Olfr553, Olfr806, Palld, Pitpnm2os1, Psma3, Rpl17-ps8, Rragd, Scarletltr, Serpina4-ps1, Shq1, Snhg7os, Snora47, Sp2, Tas2r113, Thpo, Tjap1, Uqcrb, Vmn1r192, Vmn1r46, Wfdc13, Zbtb7c |
| Cluster 3 (42 genes) |
| A530030E21Rik, ATP8, Asb18, Bst1, Cd209g, Cd9, Egfr, Eif4a2, Fabp5, Gde1, Gm4832, Gpc3, Hhat, Igfbp6, Il15ra, Il21r, Itgam, LOC545966, Lrig2, March1, Mif, Msln, Mup-ps12, Mup17, Mup6, ND2, ND3, Nrp1, Pnldc1, Ptgis, Rfc5, Rpl13, Rpl34-ps1, Rps24, Serpina9, Siglech, Slc25a30, Slpi, Smg5, Socs2, Tgfb2, Zfp663 |
| Cluster 4 (149 genes) |
| 1110065P20Rik, 1810011H11Rik, 3010003L21Rik, 6330403L08Rik, Actb, Adam32, Aif1, Aldoa, Anln, Ap1s2, Armcx3, Armcx4, Asf1b, Atf4, Atf5, Aurka, Birc5, Cad, Cbr3, Ccdc149, Ccnb2, Cd164, Cd44, Cd5l, Cdc20, Cdca3, Cdca8, Cdk2ap2, Clec4a3, Cln8, Csf1r, Ctps, Ddit3, Ddx17, Ddx39b, Dpp3, Dusp8, Ehd1, Endod1, Entpd1, Epb4.1l2, Erdr1, Evi5l, Fam195b, Fcgr4, Fgf21, Fkbp1a, Fyb, Gabarap, Gale, Gdf15, Gfpt1, Gins2, Gm10734, Gm11223, Gm34786, Gm36393, Gm5150, Gmppa, Gmppb, Gnb2, Gramd1c, Hck, Hcls1, Hexa, Hist1h1c, Hk3, Hnrnpa3, Hnrnph1, Hspa5, Hspb1, Ifrd1, Ikbip, Incenp, Inpp5d, Itgal, Jund, Lbh, Lpl, Lysmd2, Marco, Mb21d1, Mbd1, Mcm2, Mcm3, Mcm5, Mcm6, Mettl1, Mki67, Mmadhc, Mogs, Morf4l2, Myc, Ncapg2, Ncaph, Neurl1b, Nme6, Pisd-ps3, Pkmyt1, Plk3, Preb, Rasgef1b, Rbl1, Rcc2, Rcn1, Renbp, Rhob, Rhox5, Ripk3, Rnpepl1, Samhd1, Sec61a1, Sec61g, Serp1, Serpinb6c, Shtn1, Slc39a7, Slc41a3, Slc7a1, Slc7a8, Slc9a8, Smc2, Smoc2, Spats2l, Spic, Srp54c, Ssr1, Stbd1, Stmn1, Stmn1-rs1, Stx4a, Tacc3, Tmed3, Trib3, Trim25, Tuba1b, Tuba1c, Tubb4b, Tubb5, Ubb, Ube2t, Ucp2, Ucp3, Usb1, Vimp, Yipf5, Zdhhc18, Zfp622, Zpr1 |
| Cluster 5 (138 genes) |
| 2010003K11Rik, Abcb4, Abcg1, Adamts14, Adamts4, Adrbk2, Agpat9, Amz1, Anxa5, Apoa4, Arhgdia, Arhgef2, Atp6v1b2, Atpif1, Axl, Bcl6b, C1qtnf6, Camk2n2, Ccdc88b, Ccl5, Ccr7, Cd274, Cd72, Cdt1, Cenpq, Cgref1, Chpf, Clec12a, Clec4n, Csrp1, Ctsd, Cttnbp2nl, Cxcl10, Cxcl14, Cxcl9, Dfna5, Dnmt3a, Dusp2, Dusp5, Ear10, Ear12, Ear2, Ece2, Egfl7, Eif4ebp1, Eif6, Entpd2, Epdr1, Evl, Gas2l1, Gbp2, Gbp6, Gbp9, Gm13889, Gm30948, Gpsm3, Gss, H2-DMb1, Haus8, Hk3, Hmga1, Icam1, Il7r, Itm2c, Kctd17, Krt18, Krt8, Lgmn, Lmna, Maged2, Map3k14, Map7d1, Marcksl1, Mefv, Mesdc1, Mlkl, Mpeg1, Ms4a6b, Myo9b, Nfkb2, Nfkbie, Nudt18, Panx1, Parp8, Pck2, Pdgfa, Pdgfb, Pdlim7, Plekhm2, Plvap, Plxnd1, Psd4, Ptma, Ptpn1, Pwwp2b, Rab3il1, Rasa4, Rasip1, Rdh9, Rela, Relb, Rhbdf2, Rnase2a, Rnu3b1, Sars, Scamp4, Scand1, Sema3f, Shisa5, Sirpa, Slamf8, Slc12a4, Slc13a5, Slc15a3, Slc22a1, Slc43a2, Slc6a8, Slc7a7, Smox, Sod3, Sorl1, Sox4, Sqstm1, Srxn1, Tbc1d9, Tgm2, Tgtp2, Themis2, Tmem206, Tmem86a, Tnip1, Tpm1, Treml4, Uap1l1, Ubd, Vapa, Wdr92, Ywhah |
| Cluster 6 (24 genes) |
| 4922502H24Rik, 9330182O14Rik, A4galt, Astl, BC049265, C230035I16Rik, Cdh16, Gm1070, Krt6a, LOC105243223, Lrrc2, Nfatc2ip, Olfr398, Olfr6, Olfr930, Pex6, Pvalb, Pygm, Rsg1, St6galnac2, Trim45, Veph1, Zbtb7c, Zfp367 |
| Cluster 7 (114 genes) |
| 4931408D14Rik, A130072N09Rik, A430078G23Rik, Abcd2, Acvr1b, Adcy9, Adora1, Alas1, Ang6, Apol9a, Apol9b, Arid5b, Arsa, Asns, B930025P03Rik, C3, C77080, Ccdc85b, Ccne1, Cdk5rap1, Clec2h, Clec4f, Cpt1a, Cry2, Cyp39a1, D230034L24Rik, Dbp, Dedd2, Derl3, Dnajb11, E330037I15Rik, Eno1, Enpp2, Esr1, Fam134b, Fastkd1, Fbxo21, Fbxo31, Fkbp5, Folr2, Gabarapl1, Glul, Gm10804, Gm42035, Grasp, Grhl1, Gsta3, Gstt2, Iffo2, Ifi27, Ifit3, Ifit3b, Inf2, Inhbe, Klf13, Lbp, Ldlr, Meg3, Mfsd7c, Midn, Mt1, Mt2, Mtss1, Myo18a, Nceh1, Nlrp12, Nmrk1, Nr1d2, Nrg4, Nrn1, Oas1a, Oas1f, Olig1, Pcsk4, Pde6c, Per2, Pex11a, Pip5k1a, Pitpnm2, Plekhg5, Plekhg6, Plin4, Pnpla2, Por, Ppara, Ppargc1b, Prok1, Ptp4a1, Rhbg, Rnf43, Sbk1, Sgk2, Sipa1l2, Slc16a5, Slc22a7, Slc25a48, Slc2a5, Slc5a6, Sorbs3, Sp5, Stab2, Sult5a1, Tbc1d16, Tbc1d30, Tef, Tgm1, Tns2, Tox, Ugcg, Usp2, Wee1, Zfp467, Zfp703, Zfp710 |

Supplementary Table 2 Terms obtained by DAVID enrichment analysis. GO terms with Benjamini-Hochberg FDR-corrected p-values of less than 0.05 are listed

| Category | Term | p-value | p-value(BH)^a^ |
| --- | --- | --- | --- |
| cluster1 | | | |
| GOTERM_BP_3 | GO:0006629 lipid metabolic process | 9.06E-16 | 5.69E-13 |
| GOTERM_BP_3 | GO:0044281 small molecule metabolic process | 5.56E-14 | 1.78E-11 |
| GOTERM_BP_3 | GO:0055114 oxidation-reduction process | 7.19E-13 | 1.54E-10 |
| GOTERM_BP_3 | GO:0006082 organic acid metabolic process | 9.37E-12 | 1.50E-09 |
| GOTERM_BP_4 | GO:0006082 organic acid metabolic process | 8.75E-12 | 5.67E-09 |
| GOTERM_BP_2 | GO:0044710 single-organism metabolic process | 4.12E-11 | 5.68E-09 |
| GOTERM_BP_4 | GO:0043436 oxoacid metabolic process | 7.75E-12 | 1.00E-08 |
| GOTERM_BP_5 | GO:0043436 oxoacid metabolic process | 1.01E-11 | 1.64E-08 |
| GOTERM_BP_5 | GO:0019752 carboxylic acid metabolic process | 5.97E-11 | 4.86E-08 |
| GOTERM_BP_4 | GO:0008202 steroid metabolic process | 2.03E-10 | 8.77E-08 |
| GOTERM_BP_5 | GO:0006694 steroid biosynthetic process | 4.95E-10 | 2.69E-07 |
| GOTERM_BP_3 | GO:0044255 cellular lipid metabolic process | 4.87E-09 | 6.24E-07 |
| GOTERM_BP_4 | GO:0006631 fatty acid metabolic process | 2.08E-09 | 6.73E-07 |
| GOTERM_BP_5 | GO:0006631 fatty acid metabolic process | 2.48E-09 | 1.01E-06 |
| GOTERM_BP_4 | GO:0044255 cellular lipid metabolic process | 4.63E-09 | 1.20E-06 |
| GOTERM_BP_4 | GO:0008610 lipid biosynthetic process | 7.93E-09 | 1.71E-06 |
| GOTERM_BP_3 | GO:0044711 single-organism biosynthetic process | 4.82E-07 | 5.14E-05 |
| GOTERM_BP_5 | GO:0001676 long-chain fatty acid metabolic process | 1.17E-06 | 3.83E-04 |
| GOTERM_BP_4 | GO:0014070 response to organic cyclic compound | 9.80E-06 | 1.81E-03 |
| GOTERM_BP_3 | GO:0009725 response to hormone | 1.85E-04 | 1.68E-02 |
| GOTERM_BP_4 | GO:0009725 response to hormone | 1.82E-04 | 2.90E-02 |
| GOTERM_BP_4 | GO:0032870 cellular response to hormone stimulus | 2.41E-04 | 3.41E-02 |
| GOTERM_BP_5 | GO:1901655 cellular response to ketone | 1.83E-04 | 4.84E-02 |
| cluster4 | | | |
| GOTERM_BP_3 | GO:0007049 cell cycle | 3.98E-09 | 2.48E-06 |
| GOTERM_BP_2 | GO:0022402 cell cycle process | 5.58E-08 | 7.93E-06 |
| GOTERM_BP_4 | GO:0022402 cell cycle process | 5.67E-08 | 7.15E-05 |
| GOTERM_BP_3 | GO:1903047 mitotic cell cycle process | 2.28E-06 | 4.74E-04 |
| GOTERM_BP_3 | GO:0051301 cell division | 1.93E-06 | 6.03E-04 |
| GOTERM_BP_1 | GO:0051179 localization | 6.18E-05 | 7.41E-04 |
| GOTERM_BP_1 | GO:0071840 cellular component organization or biogenesis | 3.43E-05 | 8.22E-04 |
| GOTERM_BP_4 | GO:0000278 mitotic cell cycle | 2.92E-06 | 1.84E-03 |
| GOTERM_BP_2 | GO:0016043 cellular component organization | 3.01E-05 | 2.13E-03 |
| GOTERM_BP_3 | GO:0010941 regulation of cell death | 2.12E-05 | 3.30E-03 |
| GOTERM_BP_4 | GO:0007067 mitotic nuclear division | 8.89E-06 | 3.73E-03 |
| GOTERM_BP_4 | GO:0043067 regulation of programmed cell death | 1.61E-05 | 4.05E-03 |
| GOTERM_BP_4 | GO:0012501 programmed cell death | 1.45E-05 | 4.57E-03 |
| GOTERM_BP_3 | GO:0008219 cell death | 5.15E-05 | 5.34E-03 |
| GOTERM_BP_5 | GO:1903047 mitotic cell cycle process | 3.31E-06 | 5.47E-03 |
| GOTERM_BP_3 | GO:0006996 organelle organization | 4.53E-05 | 5.64E-03 |
| GOTERM_BP_4 | GO:0048285 organelle fission | 3.21E-05 | 6.73E-03 |
| GOTERM_BP_2 | GO:0006950 response to stress | 2.44E-04 | 8.63E-03 |
| GOTERM_BP_2 | GO:0044763 single-organism cellular process | 1.85E-04 | 8.72E-03 |
| GOTERM_BP_5 | GO:0007067 mitotic nuclear division | 1.15E-05 | 9.47E-03 |
| GOTERM_BP_5 | GO:0043067 regulation of programmed cell death | 2.44E-05 | 1.01E-02 |
| GOTERM_BP_4 | GO:0006986 response to unfolded protein | 5.96E-05 | 1.07E-02 |
| GOTERM_BP_5 | GO:0000280 nuclear division | 1.95E-05 | 1.07E-02 |
| GOTERM_BP_3 | GO:0035966 response to topologically incorrect protein | 1.39E-04 | 1.23E-02 |
| GOTERM_BP_2 | GO:0033036 macromolecule localization | 4.73E-04 | 1.34E-02 |
| GOTERM_BP_5 | GO:0006270 DNA replication initiation | 4.14E-05 | 1.36E-02 |
| GOTERM_BP_3 | GO:0006979 response to oxidative stress | 1.81E-04 | 1.40E-02 |
| GOTERM_BP_2 | GO:0051641 cellular localization | 6.55E-04 | 1.54E-02 |
| GOTERM_BP_5 | GO:0042981 regulation of apoptotic process | 5.78E-05 | 1.58E-02 |
| GOTERM_BP_5 | GO:0006986 response to unfolded protein | 6.80E-05 | 1.60E-02 |
| GOTERM_BP_4 | GO:0030968 endoplasmic reticulum unfolded protein response | 1.06E-04 | 1.66E-02 |
| GOTERM_BP_2 | GO:0009056 catabolic process | 9.90E-04 | 1.74E-02 |
| GOTERM_BP_2 | GO:0051234 establishment of localization | 9.00E-04 | 1.81E-02 |
| GOTERM_BP_4 | GO:0035966 response to topologically incorrect protein | 1.41E-04 | 1.96E-02 |
| GOTERM_BP_5 | GO:0030968 endoplasmic reticulum unfolded protein response | 1.19E-04 | 2.43E-02 |
| GOTERM_BP_5 | GO:0034620 cellular response to unfolded protein | 1.37E-04 | 2.49E-02 |
| GOTERM_BP_3 | GO:1901575 organic substance catabolic process | 4.67E-04 | 2.61E-02 |
| GOTERM_BP_3 | GO:0008104 protein localization | 4.39E-04 | 2.71E-02 |
| GOTERM_BP_3 | GO:0060548 negative regulation of cell death | 3.98E-04 | 2.72E-02 |
| GOTERM_BP_5 | GO:0006915 apoptotic process | 1.67E-04 | 2.73E-02 |
| GOTERM_BP_5 | GO:0097193 intrinsic apoptotic signaling pathway | 1.91E-04 | 2.84E-02 |
| GOTERM_BP_3 | GO:0007017 microtubule-based process | 6.40E-04 | 3.27E-02 |
| GOTERM_BP_4 | GO:0035967cellular response to topologically incorrect protein | 2.96E-04 | 3.67E-02 |
| GOTERM_BP_3 | GO:0070727 cellular macromolecule localization | 9.13E-04 | 3.73E-02 |
| GOTERM_BP_5 | GO:0009142 nucleoside triphosphate biosynthetic process | 2.78E-04 | 3.77E-02 |
| GOTERM_BP_3 | GO:1902589 single-organism organelle organization | 8.91E-04 | 3.89E-02 |
| GOTERM_BP_3 | GO:0051726 regulation of cell cycle | 8.33E-04 | 3.92E-02 |
| GOTERM_BP_5 | GO:0035967cellular response to topologically incorrect protein | 3.31E-04 | 4.13E-02 |
| GOTERM_BP_4 | GO:0060548 negative regulation of cell death | 4.11E-04 | 4.23E-02 |
| GOTERM_BP_2 | GO:0032879 regulation of localization | 2.80E-03 | 4.32E-02 |
| GOTERM_BP_4 | GO:0043069 negative regulation of programmed cell death | 4.09E-04 | 4.58E-02 |
| GOTERM_BP_1 | GO:0044699 single-organism process | 6.33E-03 | 4.95E-02 |
| cluster5 | | | |
| GOTERM_BP_1 | GO:0002376 immune system process | 1.85E-09 | 4.08E-08 |
| GOTERM_BP_4 | GO:0034097 response to cytokine | 7.76E-10 | 1.03E-06 |
| GOTERM_BP_2 | GO:0006955 immune response | 1.16E-07 | 1.58E-05 |
| GOTERM_BP_2 | GO:0009605 response to external stimulus | 4.85E-07 | 1.66E-05 |
| GOTERM_BP_2 | GO:0048583 regulation of response to stimulus | 6.71E-07 | 1.84E-05 |
| GOTERM_BP_2 | GO:0040012 regulation of locomotion | 4.41E-07 | 2.01E-05 |
| GOTERM_BP_2 | GO:0042221 response to chemical | 3.93E-07 | 2.69E-05 |
| GOTERM_BP_2 | GO:0006950 response to stress | 1.26E-06 | 2.87E-05 |
| GOTERM_BP_2 | GO:0032879 regulation of localization | 4.66E-06 | 9.11E-05 |
| GOTERM_BP_3 | GO:2000145 regulation of cell motility | 2.37E-07 | 1.49E-04 |
| GOTERM_BP_2 | GO:0048522 positive regulation of cellular process | 1.10E-05 | 1.67E-04 |
| GOTERM_BP_2 | GO:0048523 negative regulation of cellular process | 1.06E-05 | 1.81E-04 |
| GOTERM_BP_4 | GO:2000145 regulation of cell motility | 2.79E-07 | 1.86E-04 |
| GOTERM_BP_4 | GO:0030334 regulation of cell migration | 5.66E-07 | 2.51E-04 |
| GOTERM_BP_3 | GO:0051270 regulation of cellular component movement | 8.18E-07 | 2.58E-04 |
| GOTERM_BP_2 | GO:0048870 cell motility | 2.40E-05 | 3.29E-04 |
| GOTERM_BP_2 | GO:0051674 localization of cell | 2.40E-05 | 3.29E-04 |
| GOTERM_BP_5 | GO:0071345 cellular response to cytokine stimulus | 6.60E-07 | 3.90E-04 |
| GOTERM_BP_5 | GO:2000145 regulation of cell motility | 2.30E-07 | 4.07E-04 |
| GOTERM_BP_5 | GO:0030334 regulation of cell migration | 4.71E-07 | 4.17E-04 |
| GOTERM_BP_2 | GO:0050900 leukocyte migration | 3.50E-05 | 4.36E-04 |
| GOTERM_BP_2 | GO:0048519 negative regulation of biological process | 4.09E-05 | 4.67E-04 |
| GOTERM_BP_2 | GO:0048518 positive regulation of biological process | 4.75E-05 | 5.01E-04 |
| GOTERM_BP_2 | GO:0002684 positive regulation of immune system process | 6.11E-05 | 5.58E-04 |
| GOTERM_BP_2 | GO:0042330 taxis | 6.07E-05 | 5.94E-04 |
| GOTERM_BP_2 | GO:0002682 regulation of immune system process | 7.09E-05 | 6.07E-04 |
| GOTERM_BP_1 | GO:0040011 locomotion | 5.76E-05 | 6.33E-04 |
| GOTERM_BP_1 | GO:0051179 localization | 1.18E-04 | 6.52E-04 |
| GOTERM_BP_1 | GO:0050896 response to stimulus | 9.90E-05 | 7.26E-04 |
| GOTERM_BP_2 | GO:0023051 regulation of signaling | 1.14E-04 | 9.15E-04 |
| GOTERM_BP_2 | GO:0044406 adhesion of symbiont to host | 1.23E-04 | 9.37E-04 |
| GOTERM_BP_2 | GO:0051707 response to other organism | 1.49E-04 | 1.07E-03 |
| GOTERM_BP_3 | GO:0048522 positive regulation of cellular process | 1.37E-05 | 1.23E-03 |
| GOTERM_BP_3 | GO:0006952 defense response | 1.34E-05 | 1.41E-03 |
| GOTERM_BP_3 | GO:0002687 positive regulation of leukocyte migration | 2.71E-05 | 1.56E-03 |
| GOTERM_BP_3 | GO:0002685 regulation of leukocyte migration | 2.07E-05 | 1.63E-03 |
| GOTERM_BP_3 | GO:0050920 regulation of chemotaxis | 7.76E-06 | 1.63E-03 |
| GOTERM_BP_3 | GO:0048523 negative regulation of cellular process | 1.30E-05 | 1.64E-03 |
| GOTERM_BP_3 | GO:0048870 cell motility | 2.68E-05 | 1.69E-03 |
| GOTERM_BP_3 | GO:0070887 cellular response to chemical stimulus | 1.09E-05 | 1.71E-03 |
| GOTERM_BP_3 | GO:0045087 innate immune response | 2.59E-05 | 1.82E-03 |
| GOTERM_BP_3 | GO:1901700 response to oxygen-containing compound | 3.56E-05 | 1.87E-03 |
| GOTERM_BP_2 | GO:0009607 response to biotic stimulus | 2.89E-04 | 1.98E-03 |
| GOTERM_BP_3 | GO:0016477 cell migration | 4.36E-05 | 2.11E-03 |
| GOTERM_BP_2 | GO:0040017 positive regulation of locomotion | 3.24E-04 | 2.11E-03 |
| GOTERM_BP_3 | GO:0002684 positive regulation of immune system process | 6.64E-05 | 2.33E-03 |
| GOTERM_BP_3 | GO:0009966 regulation of signal transduction | 5.59E-05 | 2.35E-03 |
| GOTERM_BP_3 | GO:0030595 leukocyte chemotaxis | 5.25E-05 | 2.36E-03 |
| GOTERM_BP_3 | GO:0042330 taxis | 6.51E-05 | 2.42E-03 |
| GOTERM_BP_3 | GO:0006935 chemotaxis | 6.27E-05 | 2.47E-03 |
| GOTERM_BP_2 | GO:0016043 cellular component organization | 4.47E-04 | 2.78E-03 |
| GOTERM_BP_4 | GO:0050920 regulation of chemotaxis | 8.46E-06 | 2.81E-03 |
| GOTERM_BP_3 | GO:0010646 regulation of cell communication | 1.11E-04 | 3.68E-03 |
| GOTERM_BP_1 | GO:0071840 cellular component organization or biogenesis | 9.06E-04 | 3.98E-03 |
| GOTERM_BP_3 | GO:0043207 response to external biotic stimulus | 1.65E-04 | 5.18E-03 |
| GOTERM_BP_2 | GO:0048584 positive regulation of response to stimulus | 9.05E-04 | 5.38E-03 |
| GOTERM_BP_3 | GO:0010033 response to organic substance | 1.82E-04 | 5.45E-03 |
| GOTERM_BP_3 | GO:0032101 regulation of response to external stimulus | 1.93E-04 | 5.51E-03 |
| GOTERM_BP_4 | GO:1902531 regulation of intracellular signal transduction | 3.52E-05 | 5.84E-03 |
| GOTERM_BP_4 | GO:0048870 cell motility | 3.16E-05 | 5.99E-03 |
| GOTERM_BP_4 | GO:0050900 leukocyte migration | 4.07E-05 | 5.99E-03 |
| GOTERM_BP_5 | GO:1902531 regulation of intracellular signal transduction | 2.86E-05 | 6.32E-03 |
| GOTERM_BP_2 | GO:0051239 regulation of multicellular organismal process | 1.18E-03 | 6.46E-03 |
| GOTERM_BP_2 | GO:0044419 interspecies interaction between organisms | 1.13E-03 | 6.46E-03 |
| GOTERM_BP_4 | GO:0045087 innate immune response | 2.93E-05 | 6.47E-03 |
| GOTERM_BP_1 | GO:0022610 biological adhesion | 1.79E-03 | 6.55E-03 |
| GOTERM_BP_4 | GO:0016477 cell migration | 5.07E-05 | 6.72E-03 |
| GOTERM_BP_5 | GO:0002687 positive regulation of leukocyte migration | 2.71E-05 | 6.84E-03 |
| GOTERM_BP_2 | GO:0051716 cellular response to stimulus | 1.32E-03 | 6.96E-03 |
| GOTERM_BP_3 | GO:2000147 positive regulation of cell motility | 2.72E-04 | 7.12E-03 |
| GOTERM_BP_4 | GO:0006935 chemotaxis | 6.99E-05 | 7.12E-03 |
| GOTERM_BP_3 | GO:0050921 positive regulation of chemotaxis | 2.62E-04 | 7.18E-03 |
| GOTERM_BP_5 | GO:0050900 leukocyte migration | 3.71E-05 | 7.26E-03 |
| GOTERM_BP_5 | GO:0002685 regulation of leukocyte migration | 2.07E-05 | 7.29E-03 |
| GOTERM_BP_5 | GO:0034612 response to tumor necrosis factor | 1.67E-05 | 7.36E-03 |
| GOTERM_BP_5 | GO:0007249 I-kappaB kinase/NF-kappaB signaling | 2.54E-05 | 7.48E-03 |
| GOTERM_BP_5 | GO:0016477 cell migration | 4.26E-05 | 7.51E-03 |
| GOTERM_BP_4 | GO:0006954 inflammatory response | 6.91E-05 | 7.63E-03 |
| GOTERM_BP_4 | GO:0002687 positive regulation of leukocyte migration | 2.91E-05 | 7.70E-03 |
| GOTERM_BP_5 | GO:0009966 regulation of signal transduction | 5.29E-05 | 7.78E-03 |
| GOTERM_BP_4 | GO:0034341 response to interferon-gamma | 8.35E-05 | 7.91E-03 |
| GOTERM_BP_4 | GO:0009966 regulation of signal transduction | 6.86E-05 | 8.26E-03 |
| GOTERM_BP_3 | GO:0040017 positive regulation of locomotion | 3.44E-04 | 8.32E-03 |
| GOTERM_BP_3 | GO:0051272 positive regulation of cellular component movement | 3.32E-04 | 8.34E-03 |
| GOTERM_BP_5 | GO:0030595 leukocyte chemotaxis | 5.25E-05 | 8.41E-03 |
| GOTERM_BP_2 | GO:0051241 negative regulation of multicellular organismal process | 1.67E-03 | 8.46E-03 |
| GOTERM_BP_4 | GO:0002688 regulation of leukocyte chemotaxis | 9.76E-05 | 8.61E-03 |
| GOTERM_BP_2 | GO:0023056 positive regulation of signaling | 2.01E-03 | 9.81E-03 |
| GOTERM_BP_3 | GO:0030029 actin filament-based process | 4.54E-04 | 1.02E-02 |
| GOTERM_BP_3 | GO:0032103 positive regulation of response to external stimulus | 4.71E-04 | 1.02E-02 |
| GOTERM_BP_3 | GO:0001775 cell activation | 4.53E-04 | 1.05E-02 |
| GOTERM_BP_5 | GO:0034341 response to interferon-gamma | 7.88E-05 | 1.07E-02 |
| GOTERM_BP_4 | GO:0044406 adhesion of symbiont to host | 1.30E-04 | 1.07E-02 |
| GOTERM_BP_2 | GO:0051234 establishment of localization | 2.40E-03 | 1.13E-02 |
| GOTERM_BP_5 | GO:0002688 regulation of leukocyte chemotaxis | 9.20E-05 | 1.16E-02 |
| GOTERM_BP_3 | GO:0009617 response to bacterium | 5.69E-04 | 1.19E-02 |
| GOTERM_BP_2 | GO:0051050 positive regulation of transport | 2.74E-03 | 1.20E-02 |
| GOTERM_BP_2 | GO:1902578 single-organism localization | 2.72E-03 | 1.24E-02 |
| GOTERM_BP_5 | GO:0043122 regulation of I-kappaB kinase/NF-kappaB signaling | 1.10E-04 | 1.30E-02 |
| GOTERM_BP_2 | GO:0019882 antigen processing and presentation | 3.11E-03 | 1.32E-02 |
| GOTERM_BP_4 | GO:0051707 response to other organism | 1.85E-04 | 1.44E-02 |
| GOTERM_BP_2 | GO:0048585 negative regulation of response to stimulus | 3.51E-03 | 1.45E-02 |
| GOTERM_BP_4 | GO:0030335 positive regulation of cell migration | 2.24E-04 | 1.64E-02 |
| GOTERM_BP_2 | GO:0050793 regulation of developmental process | 4.31E-03 | 1.72E-02 |
| GOTERM_BP_4 | GO:1901701 cellular response to oxygen-containing compound | 2.82E-04 | 1.86E-02 |
| GOTERM_BP_4 | GO:2000147 positive regulation of cell motility | 2.97E-04 | 1.87E-02 |
| GOTERM_BP_4 | GO:0050921 positive regulation of chemotaxis | 2.78E-04 | 1.93E-02 |
| GOTERM_BP_3 | GO:0006928 movement of cell or subcellular component | 9.99E-04 | 1.95E-02 |
| GOTERM_BP_4 | GO:0002690 positive regulation of leukocyte chemotaxis | 3.28E-04 | 1.96E-02 |
| GOTERM_BP_4 | GO:0030036 actin cytoskeleton organization | 4.04E-04 | 1.97E-02 |
| GOTERM_BP_4 | GO:0030168 platelet activation | 4.23E-04 | 1.99E-02 |
| GOTERM_BP_2 | GO:0044707 single-multicellular organism process | 5.14E-03 | 2.00E-02 |
| GOTERM_BP_3 | GO:0048584 positive regulation of response to stimulus | 9.99E-04 | 2.01E-02 |
| GOTERM_BP_4 | GO:0060326 cell chemotaxis | 4.04E-04 | 2.04E-02 |
| GOTERM_BP_4 | GO:0051272 positive regulation of cellular component movement | 3.63E-04 | 2.08E-02 |
| GOTERM_BP_4 | GO:0035556 intracellular signal transduction | 4.58E-04 | 2.08E-02 |
| GOTERM_BP_4 | GO:1902533 positive regulation of intracellular signal transduction | 3.96E-04 | 2.09E-02 |
| GOTERM_BP_5 | GO:0051259 protein oligomerization | 2.07E-04 | 2.13E-02 |
| GOTERM_BP_4 | GO:0032496 response to lipopolysaccharide | 3.92E-04 | 2.15E-02 |
| GOTERM_BP_4 | GO:1902533 positive regulation of intracellular signal transduction | 5.28E-04 | 2.17E-02 |
| GOTERM_BP_4 | GO:0002237 response to molecule of bacterial origin | 5.13E-04 | 2.18E-02 |
| GOTERM_BP_4 | GO:0032103 positive regulation of response to external stimulus | 5.05E-04 | 2.22E-02 |
| GOTERM_BP_5 | GO:0030335 positive regulation of cell migration | 2.04E-04 | 2.23E-02 |
| GOTERM_BP_3 | GO:0044403 symbiosis, encompassing mutualism through parasitism | 1.20E-03 | 2.28E-02 |
| GOTERM_BP_3 | GO:0010035 response to inorganic substance | 1.32E-03 | 2.35E-02 |
| GOTERM_BP_4 | GO:0002548 monocyte chemotaxis | 5.99E-04 | 2.39E-02 |
| GOTERM_BP_3 | GO:0009967 positive regulation of signal transduction | 1.30E-03 | 2.39E-02 |
| GOTERM_BP_3 | GO:0008219 cell death | 1.51E-03 | 2.48E-02 |
| GOTERM_BP_3 | GO:0001817 regulation of cytokine production | 1.43E-03 | 2.48E-02 |
| GOTERM_BP_5 | GO:2000147 positive regulation of cell motility | 2.71E-04 | 2.49E-02 |
| GOTERM_BP_5 | GO:0060326 cell chemotaxis | 3.76E-04 | 2.53E-02 |
| GOTERM_BP_3 | GO:0001562 response to protozoan | 1.51E-03 | 2.54E-02 |
| GOTERM_BP_5 | GO:0050921 positive regulation of chemotaxis | 2.63E-04 | 2.55E-02 |
| GOTERM_BP_5 | GO:0035556 intracellular signal transduction | 3.70E-04 | 2.59E-02 |
| GOTERM_BP_5 | GO:0030168 platelet activation | 4.03E-04 | 2.61E-02 |
| GOTERM_BP_5 | GO:0030036 actin cytoskeleton organization | 3.66E-04 | 2.66E-02 |
| GOTERM_BP_2 | GO:0065008 regulation of biological quality | 7.21E-03 | 2.72E-02 |
| GOTERM_BP_5 | GO:0002690 positive regulation of leukocyte chemotaxis | 3.12E-04 | 2.73E-02 |
| GOTERM_BP_5 | GO:0032496 response to lipopolysaccharide | 3.60E-04 | 2.73E-02 |
| GOTERM_BP_5 | GO:0051272 positive regulation of cellular component movement | 3.31E-04 | 2.75E-02 |
| GOTERM_BP_5 | GO:1902533 positive regulation of intracellular signal transduction | 3.49E-04 | 2.77E-02 |
| GOTERM_BP_2 | GO:0044085 cellular component biogenesis | 7.84E-03 | 2.87E-02 |
| GOTERM_BP_3 | GO:0051241 negative regulation of multicellular organismal process | 1.80E-03 | 2.88E-02 |
| GOTERM_BP_3 | GO:0023056 positive regulation of signaling | 2.19E-03 | 3.09E-02 |
| GOTERM_BP_3 | GO:0051049 regulation of transport | 2.17E-03 | 3.14E-02 |
| GOTERM_BP_3 | GO:0050817 coagulation | 2.12E-03 | 3.14E-02 |
| GOTERM_BP_3 | GO:0044765 single-organism transport | 2.10E-03 | 3.18E-02 |
| GOTERM_BP_3 | GO:0010647 positive regulation of cell communication | 2.07E-03 | 3.22E-02 |
| GOTERM_BP_3 | GO:0022607 cellular component assembly | 2.33E-03 | 3.22E-02 |
| GOTERM_BP_5 | GO:0060445 branching involved in salivary gland morphogenesis | 5.36E-04 | 3.33E-02 |
| GOTERM_BP_5 | GO:0009617 response to bacterium | 5.67E-04 | 3.40E-02 |
| GOTERM_BP_2 | GO:0040013 negative regulation of locomotion | 9.55E-03 | 3.40E-02 |
| GOTERM_BP_3 | GO:0001816 cytokine production | 2.85E-03 | 3.84E-02 |
| GOTERM_BP_3 | GO:0051050 positive regulation of transport | 2.93E-03 | 3.86E-02 |
| GOTERM_BP_2 | GO:0045785 positive regulation of cell adhesion | 1.12E-02 | 3.90E-02 |
| GOTERM_BP_5 | GO:1901623 regulation of lymphocyte chemotaxis | 6.82E-04 | 3.95E-02 |
| GOTERM_BP_4 | GO:0042832 defense response to protozoan | 1.08E-03 | 4.12E-02 |
| GOTERM_BP_4 | GO:0007166 cell surface receptor signaling pathway | 1.11E-03 | 4.13E-02 |
| GOTERM_BP_1 | GO:0023052 signaling | 1.40E-02 | 4.32E-02 |
| GOTERM_BP_2 | GO:0044700 single organism signaling | 1.30E-02 | 4.40E-02 |
| GOTERM_BP_5 | GO:0043123 positive regulation of I-kappaB kinase/NF-kappaB signaling | 8.05E-04 | 4.50E-02 |
| GOTERM_BP_3 | GO:0001819 positive regulation of cytokine production | 3.53E-03 | 4.54E-02 |
| GOTERM_BP_4 | GO:0071822 protein complex subunit organization | 1.26E-03 | 4.54E-02 |
| GOTERM_BP_4 | GO:0048246 macrophage chemotaxis | 1.42E-03 | 4.75E-02 |
| GOTERM_BP_3 | GO:0048585 negative regulation of response to stimulus | 3.78E-03 | 4.76E-02 |
| GOTERM_BP_4 | GO:0009967 positive regulation of signal transduction | 1.47E-03 | 4.79E-02 |
| GOTERM_BP_4 | GO:0007596 blood coagulation | 1.42E-03 | 4.85E-02 |
| GOTERM_BP_4 | GO:0031333 negative regulation of protein complex assembly | 1.39E-03 | 4.89E-02 |
| GOTERM_BP_5 | GO:0007166 cell surface receptor signaling pathway | 9.17E-04 | 4.95E-02 |
| GOTERM_BP_3 | GO:0030029 actin filament-based process | 4.54E-04 | 1.02E-02 |
| cluster7 | | | |
| GOTERM_BP_1 | GO:0048511 rhythmic process | 3.69E-05 | 8.11E-04 |

^a^Corrected p-value for multiple testing using the Benjamini-Hochberg method

Supplementary Table 3 Pathways obtained by DAVID enrichment analyses. Listed pathways have Benjamini-Hochberg FDR-corrected p-values of less than 0.05.

| KEGG Pathway ID: Pathway title | p-value | p-value(BH)^a^ |
| --- | --- | --- |
| Cluster 1 | | |
| mmu00140: Steroid hormone biosynthesis | 1.30E-13 | 2.19E-11 |
| mmu00830: Retinol metabolism | 5.39E-07 | 4.53E-05 |
| mmu04750: Inflammatory mediator regulation of TRP channels | 7.56E-06 | 4.23E-04 |
| mmu01100: Metabolic pathways | 3.01E-05 | 1.26E-03 |
| mmu04913: Ovarian steroidogenesis | 9.47E-05 | 3.18E-03 |
| mmu00120: Primary bile acid biosynthesis | 2.72E-04 | 7.58E-03 |
| mmu00590: Arachidonic acid metabolism | 7.68E-04 | 1.60E-02 |
| mmu05204: Chemical carcinogenesis | 8.93E-04 | 1.65E-02 |
| mmu04726: Serotonergic synapse | 6.95E-04 | 1.66E-02 |
| mmu04976: Bile secretion | 2.64E-03 | 4.34E-02 |
| Cluster2 | | |
| mmu05010: Alzheimer's disease | 1.43E-03 | 4.01E-02 |
| mmu05016: Huntington's disease | 2.17E-03 | 4.04E-02 |
| mmu05012: Parkinson's disease | 7.54E-04 | 4.21E-02 |
| Cluster 4 | | |
| mmu00520: Amino sugar and nucleotide sugar metabolism | 5.54E-06 | 0.0008 |
| mmu04110: Cell cycle | 1.84E-05 | 0.0013 |
| mmu04145: Phagosome | 1.81E-04 | 0.0063 |
| mmu04141: Protein processing in endoplasmic reticulum | 1.60E-04 | 0.0075 |

^a^Corrected p-value for multiple testing using Benjamini-Hochberg method.
